# Both candidate gene and neutral genetic diversity correlate with parasite resistance in female Mediterranean mouflon

**DOI:** 10.1186/s12898-019-0228-x

**Published:** 2019-03-05

**Authors:** Elodie Portanier, Mathieu Garel, Sébastien Devillard, Daniel Maillard, Jocelyn Poissant, Maxime Galan, Slimania Benabed, Marie-Thérèse Poirel, Jeanne Duhayer, Christian Itty, Gilles Bourgoin

**Affiliations:** 1 Univ Lyon, Université Claude Bernard Lyon 1, CNRS, Laboratoire de Biométrie et Biologie Évolutive, 69100 Villeurbanne, France; 2 Office National de la Chasse et de la Faune Sauvage, Unité Ongulés Sauvages, 5 allée de Bethléem, Z.I. Mayencin, 38610 Gières, France; 3 Université de Lyon, VetAgro Sup, Campus Vétérinaire de Lyon, 1 Avenue Bourgelat, BP 83, 69280 Marcy l’Etoile, France; 4 Department of Ecosystem and Public Health, University of Calgary, Calgary, Canada; 5 CBGP, INRA, CIRAD, IRD, Montpellier SupAgro, Université de Montpellier, 34980 Montferrier Sur Lez, France

**Keywords:** Heterozygosity-fitness correlations, Immunocompetence, MHC, Gastro-intestinal nematodes, Coccidia

## Abstract

**Background:**

Parasite infections can have substantial impacts on population dynamics and are accordingly a key challenge for wild population management. Here we studied genetic mechanisms driving parasite resistance in a large herbivore through a comprehensive approach combining measurements of neutral (16 microsatellites) and adaptive (MHC DRB1 exon 2) genetic diversity and two types of gastrointestinal parasites (nematodes and coccidia).

**Results:**

While accounting for other extrinsic and intrinsic predictors known to impact parasite load, we show that both neutral genetic diversity and DRB1 are associated with resistance to gastrointestinal nematodes. Intermediate levels of multi-locus heterozygosity maximized nematodes resistance, suggesting that both in- and outbreeding depression might occur in the population. DRB1 heterozygosity and specific alleles effects were detected, suggesting the occurrence of heterozygote advantage, rare-allele effects and/or fluctuating selection. On the contrary, no association was detected between genetic diversity and resistance to coccidia, indicating that different parasite classes are impacted by different genetic drivers.

**Conclusions:**

This study provides important insights for large herbivores and wild sheep pathogen management, and in particular suggests that factors likely to impact genetic diversity and allelic frequencies, including global changes, are also expected to impact parasite resistance.

**Electronic supplementary material:**

The online version of this article (10.1186/s12898-019-0228-x) contains supplementary material, which is available to authorized users.

## Background

Parasites are an important component of ecosystems and can have substantial impacts on host fitness and population dynamics. Parasites can affect body condition (e.g. [1–3]), reproductive success (e.g., [4, 5]), survival (e.g., [6]), feeding behavior (e.g., [7]) and/or interspecific interactions (e.g., [8, 9]). While parasitism causes significant economic losses in animal production around the world (e.g. gastrointestinal nematodes (GINs)) [10, 11], in wild populations its impact on individual and population viability [12] can lead to management and conservation issues [13, 14].

Resistance to parasites, defined as the “*host’s ability to interact with and control the lifecycle of the parasite*” [15, 16], depends in part on the genetically determined immune system of hosts and hence involves both the genetic characteristics (e.g. presence of specific alleles) and variability of hosts [17–20]. The influence of genetics on parasite resistance is also mediated by other extrinsic and intrinsic factors such as population density, environmental conditions, age, sex and body condition [18, 20–23]. Consequently, all the elements likely to impact genetic diversity are expected to impact parasite resistance as well. In the current context of habitat fragmentation [24, 25] impacting population sizes, gene flow and thus genetic diversity [26–28] and of climate change modifying parasite environmental persistence and dynamics [29–31], gathering knowledge on the genetics of parasite resistance has become crucial for population management and conservation purposes.

A large body of literature on the genetics of parasite resistance investigates heterozygosity-fitness correlations (HFCs) using heterozygosity as a measure of genetic diversity and parasite resistance as a fitness proxy. Positive relationships between pathogen resistance and heterozygosity have been evidenced in numerous taxa (e.g. wild boars, *Sus scrofa*, [32]; raccoons, *Procyon lotor*, [33]; Alpine ibex, *Capra ibex,* [34]; mongooses, *Mungos mungo*, [35]). Effects of specific loci and especially candidate genes (i.e. encoding genes associated with immunity) on pathogen resistance have also been documented (see e.g., [36–41]). For instance, Luikart et al. [42] had shown that the link between heterozygosity and parasite burden relies on microsatellites located in candidate genes instead of on microsatellites in genome portions assumed as neutral. Although a large majority of studies evidenced positive correlations between parasite resistance and heterozygosity, contrasting results can nevertheless be observed: inconclusive studies [43], negative correlations (e.g., [44, 45]) or no correlation between pathogen resistance and heterozygosity and/or specific loci/alleles (e.g., [46–49]) can be found.

Three main hypotheses might explain HFCs [50]: (i) the *direct effect hypothesis* positing a direct link of genetic markers with fitness (e.g. encoding genes), (ii) the *local effect hypothesis* (or *indirect effect hypothesis*) claiming that the markers considered are in linkage disequilibrium (non-random association of alleles at different loci) with fitness-linked loci and (iii) the *general effect hypothesis* asserting that the heterozygote advantage is due to a genome-wide effect of fitness loci with more diverse individuals thought to be more efficient in coping with infections (e.g., [51]). However, since the existence and detection of HFCs are largely environment- and context-dependent [52], distinguishing between the three hypotheses is a challenging task. In particular, HFCs depend on the inbreeding level of the population (identity disequilibrium, [52]), the genetic markers and fitness components used and the ability of these markers to capture genome-wide diversity [53–55]. In the case of parasite resistance, HFCs may also depend on the parasites and hosts species studied (e.g., [48, 56]). Indeed, not all parasites have the same effects on hosts and thus the effects of genetic diversity on resistance may vary from one class to another and according to co-infections [33, 57]. In addition, immunocompetence of individuals is a highly polygenic trait involving numerous genes associated with immunity functions (e.g., X-chromosome [58]; gamma interferon [59]; Toll-like receptors [60]; major histocompatibility complex (MHC) [61]; reviewed by [18, 20]). Comparative studies combining different approaches and different parasites types are thus needed to better understand functional links between genetics and pathogen resistance.

Here, we proposed to gain better knowledge on the genetics of resistance and underlying mechanisms by combining candidate genes and neutral diversity approaches for two parasites classes, gastrointestinal nematodes (GINs) and protozoan parasites (Coccidia, *Eimeria* spp.) in female Mediterranean mouflon (*Ovis gmelini musimon* × *Ovis* sp.). GINs and coccidia are common parasites of small ruminants [62, 63] and are known to impact fitness (e.g., [64, 65]) and cause important economic losses in domestic livestock [66, 67]. While they have been the object of numerous studies on genetic parasite resistance in domestic sheep (e.g., [68–70], see also [71] for a review), they have been much less investigated in wild sheep species (but see [36, 58, 59, 72] for examples in feral Soay sheep, *Ovis aries*, and [42] for an example in bighorn sheep, *Ovis canadensis*) despite similar expected detrimental effects and the existence, for these wild species, of both conservation (e.g., [73, 74]) and management issues (e.g., [75–77]).

In both the neutral diversity and candidate gene approaches, we first accounted for other extrinsic and intrinsic predictors known to impact parasite load (e.g., socio-spatial organization [78]; population density [22]; age, sex [18]; body condition [3]). We then assessed, for the neutral diversity approach, if multi-locus heterozygosity from a set of neutral markers (16 microsatellites) was associated with parasite resistance as measured by fecal egg or fecal oocyst counts (FEC or FOC, for GINs and coccidia, respectively). In line with most HFC studies, we expected the more heterozygous individuals to be more resistant to parasite infection because more diverse individuals are expected to carry more adaptive alleles to resist parasites and/or to less express deleterious recessive alleles (e.g., [34, 36, 79]). For the candidate gene approach, we focused on MHC DRB1 class II gene, known to encode for binding proteins presenting extracellular antigens to T-lymphocytes [80] and to be linked to parasite resistance in sheep and mammals (see e.g., [61, 68, 81]). A high variation at MHC class II loci is often considered advantageous since it should enable an increased number of pathogens to be recognized and subsequent immune response [82] (see also [83, 84] for reviews). However, the presence of certain genotypes or alleles at candidate loci has also been shown to be associated with parasite resistance or susceptibility (e.g., [69, 70]). We thus independently tested for the effects on parasite resistance of genotypes, heterozygosity and the presence of specific alleles at DRB1 locus in order to discriminate between the diverse possible effects. We expected homozygous individuals at candidate locus to be more susceptible to parasite infections while specific association with genotypes and/or alleles could also be observed. In order to disentangle between genome-wide or immune gene associations, neutral multi-locus heterozygosity and immune gene were all considered in the same analyses. Finally, since GINs and coccidia are two very different classes of parasites (macro-parasites and protozoan micro-parasites, respectively) driven by diverse immune mechanisms [85, 86], results between them were expected to be different (see e.g., [33, 87, 88]).

## Results

### Genetic diversity

The multi-locus heterozygosity sMLH ranged from 0.36 to 1.36 and had an average value of 0.91. The set of 16 microsatellites showed a *g*_*2*_ not significantly different from zero, neither when the whole population was considered (*g*_*2*_ = 0.008 ± 0.009, *p* = 0.10) nor when analyses were performed for each socio-spatial unit separately (*Nf*: *g*_*2*_ = − 0.009, *p* = 0.69; *Cf*: *g*_*2*_ = − 0.007, *p* = 0.16; *Sf*: *g*_*2*_ = 0.06, *p* = 0.07). Three DRB1 alleles, which have all been previously described in domestic sheep, were identified (*Ovar*-DRB1*0324, *Ovar*-DRB1*07012 and *Ovar*-DRB1*0114, see [89], GeneBank accession numbers: *Ovar*-DRB1 *0324, DQ659119.2, *Ovar*-DRB1 *07012, AY884017.2 and *Ovar*-DRB1*0114, DQ659116.2) leading to six different genotypes (named from *A* to *F,* see Table 1). The two individuals presenting genotype *F* were removed from the dataset before analyses to avoid false positive effects caused by a too small sample size. A total of 77 individuals representing 118 observations were thus considered in subsequent analyses.

**Table 1:** DRB1 alleles, genotypes and number of individuals in each class (*n*)

Genotype	*A*	*B*	*C*	*D*	*E*	*F*
Alleles	*0324/*0324	*0324/*07012	*0324/*0114	*07012/*07012	*07012/*0114	*0114/*0114
*n*	44	29	31	7	7	2

### Parasite prevalence and abundance

The prevalence of coccidia was 100% with FOC ranging from 25 to 11,300 OPG (median FOC = 925). GINs were present in 76 out of 77 individuals with FEC ranging from 0 to 5100 EPG (median FEC = 350). Repeated measurements were available for 29 individuals (70 observations) and mean repeatability for FOC was 0.08 ([0.00–0.44]_95%_), while it was higher for FEC with an average value of 0.41 ([0.13–0.70]_95%_).

### Non-genetic variables

For FOC, the five first models were equivalent (ΔAICc < 2) and included age, body condition, time lapse between sampling and coproscopy, and Julian date (see Additional files 1 and 2 for more details). The best non-genetic model retained for coccidia thus accounted for these four non-genetic variables. For GINs, the best non-genetic model included only the effect of body condition (see Additional files 1 and 2 for more details).

### HFC and locus-specific effects

**Fig. 1:**
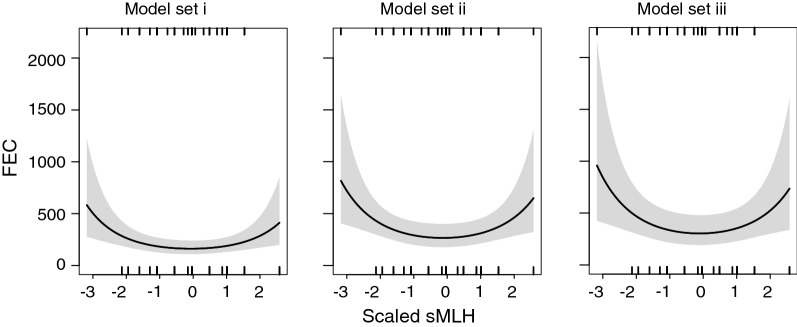
Predicted GINs burdens (FEC) values as a function of scaled sMLH from each best genetic model in each model set: (i) sMLH + DRB1 heterozygosity status, (ii) sMLH + presence of DRB1*0114 allele and (iii) sMLH + DRB1genotypes. Black lines represent predicted values and grey bands represent the 95% confidence interval. Upper and lower ticks represent the number of positive and negative residuals, respectively

In the second step of the inferential approach, we added genetic predictors to the best non-genetic models previously retained. None of the genetic predictors showed a VIF higher than three in any of the model sets for both parasite types, indicating no correlation issues (Additional file 3: Table S4). When considering coccidia, no quadratic relationship between sMLH and FOC was detected (Additional file 3: Table S5) and the best model was the non-genetic model (Table 2) indicating that the genetic predictors we studied were not significantly linked to coccidia resistance. For GINs, a quadratic relationship between sMLH and FEC was detected in the three sets of models (i) DRB1 heterozygosity status, (ii) presence of specific DRB1 alleles and (iii) DRB1genotypes (Fig. 1, Additional file 3: Table S5). In all models where sMLH and sMLH^2^ appeared, estimates were negative for sMLH and positive for sMLH^2^ (Table 3) indicating a U-shaped relationship (Fig. 1).

**Table 2:** Model selection of mixed-effects models based on corrected Akaike’s Information Criterion (AICc) for testing the effects of sMLH and DRB1 gene on parasite resistance as measured by FOC and FEC

	*d.f.*	*AICc*	*ΔAICc*	*Weight*	*Model set*
*FOC*					
*NG*	9	379.11	0.00	0.191	all
*NG* + R2	10	379.53	0.42	0.154	ii
*NG* + R1	10	380.07	0.97	0.117	ii
*NG* + HDRB	10	381.08	1.97	0.071	i
*NG* + sMLH	10	381.27	2.16	0.065	all
*NG* + R3	10	381.48	2.38	0.058	ii
*NG* + R1 + R2	11	381.61	2.51	0.054	ii
*NG* + R2 + R3	11	381.73	2.63	0.051	ii
*NG* + sMLH + R2	11	381.85	2.74	0.048	ii
*NG* + sMLH + R1	11	382.30	3.19	0.039	ii
*NG* + R1 + R3	11	382.50	3.40	0.035	ii
*NG* + sMLH + HDRB	11	383.26	4.16	0.024	i
*NG* + sMLH + R3	11	383.67	4.57	0.019	ii
*NG* + sMLH + R1 + R2	12	383.96	4.85	0.017	ii
*NG* + R1 + R2 + R3	12	384.00	4.90	0.016	ii
*NG* + sMLH + R2 + R3	12	384.06	4.96	0.016	ii
*NG* + sMLH + R1 + R3	12	384.78	5.67	0.011	ii
*NG* + G*_*DRB1	13	386.21	7.11	0.005	iii
*NG* + sMLH + R1 + R2 + R3	13	386.38	7.27	0.005	ii
*NG* + sMLH + G*_*DRB1	14	388.65	9.54	0.002	iii
*FEC*					
*NG* + sMLH + sMLH^2^ + HDRB	8	378.34	0.00	0.298	i
*NG* + sMLH + sMLH^2^ + R3	8	379.61	1.28	0.158	ii
*NG* + sMLH + sMLH^2^ + R1 + R3	9	380.61	2.27	0.096	ii
*NG* + sMLH + sMLH^2^ + R1 + R2 + R3	10	381.07	2.73	0.076	ii
*NG* + R1 + R3	7	381.52	3.18	0.061	ii
*NG* + R3	6	381.61	3.28	0.058	ii
*NG* + sMLH + sMLH^2^ + R2 + R3	9	381.65	3.31	0.057	ii
*NG* + HDRB	6	382.33	3.99	0.041	i
*NG* + R1 + R2 + R3	8	382.44	4.11	0.038	ii
*NG* + sMLH + sMLH^2^	7	382.90	4.56	0.030	all
*NG* + sMLH + sMLH^2^ + G_ DRB1	11	383.27	4.93	0.025	iii
*NG* + R2 + R3	7	383.85	5.51	0.019	ii
*NG* + G*_*DRB1	9	384.73	6.40	0.012	iii
*NG* + sMLH + sMLH^2^ + R1	8	384.87	6.54	0.011	ii
*NG* + sMLH + sMLH^2^ + R2	8	385.19	6.85	0.010	ii
*NG* + sMLH + sMLH^2^ + R1 + R2	9	387.04	8.70	0.004	ii
*NG*	5	387.61	9.27	0.003	all
*NG* + R1	6	389.07	10.73	0.001	ii
*NG* + R2	6	389.63	11.30	0.001	ii
*NG* + R1 + R2	7	391.33	12.99	0.000	ii

Three sets of genetic models have been tested on FOC and FEC including either (i) the effects of sMLH and DRB1 heterozygosity status (HDRB), (ii) the effects of sMLH and the presence of specific DRB1 alleles or (iii) the effects of sMLH and DRB1genotypes (G_DRB1)*. d.f.* are the degree of freedom, *weight* is the Akaike weight. *NG* stands for the non-genetic variables retained from the first step of the modeling approach (see Additional file 1). R1, R2 and R3 stand for DRB1 *0324, DRB1*07012 * and DRB1*0114 alleles, respectively

**Table 3:** Model estimates and goodness of fit (R^2^c and R^2^m) of the best genetic model for model sets (i) testing the effects of sMLH and DRB1 heterozygosity status (HDRB), (ii) testing the effects of sMLH and the presence of specific DRB1 alleles and (iii) testing the effects of MLH and DRB1genotypes (G_DRB1) on FEC

	β ± SE	*t* value	*p*	*R* ^*2*^ *c*	*R* ^*2*^ *m*
*Model set (i)*				0.44	0.27
Intercept	6.10 ± 0.17				
Body condition	− 0.48 ± 0.11	− 4.24	***		
sMLH	− 1.01 ± 1.23	− 0.82			
sMLH^2^	3.36 ± 1.19	2.80	**		
HDRB	− 0.61 ± 0.24	− 2.60	*		
*Model set (ii)*				0.45	0.28
Intercept	5.95 ± 0.14				
Body condition	− 0.51 ± 0.11	− 4.49	***		
sMLH	− 0.67 ± 1.26	− 0.54			
sMLH^2^	3.07 ± 1.23	2.50	*		
DRB1*0114	− 0.63 ± 0.27	− 2.33	*		
*Model set (iii)*				0.46	0.28
Intercept	6.08 ± 0.19				
Body condition	− 0.49 ± 0.12	− 4.28	***		
sMLH	− 0.77 ± 1.26	− 0.61			
sMLH^2^	3.10 ± 1.30	2.39	*		
G_DRB1 B	− 0.41 ± 0.30	− 1.38			
G_DRB1 C	− 0.87 ± 0.31	− 2.77	**		
G_DRB1 D	0.14 ± 0.53	0.27			
G_DRB1 E	− 0.39 ± 0.51	− 0.76			

sMLH is the standardized multilocus heterozygosity. Non-genetic terms were retained in the first step of the modeling approach (see main text). *P*-*values* are coded by asterisks: “***” for *p* < 0.001, “**” for *p* < 0.01, “*” for *p* < 0.05

**Fig. 2:**
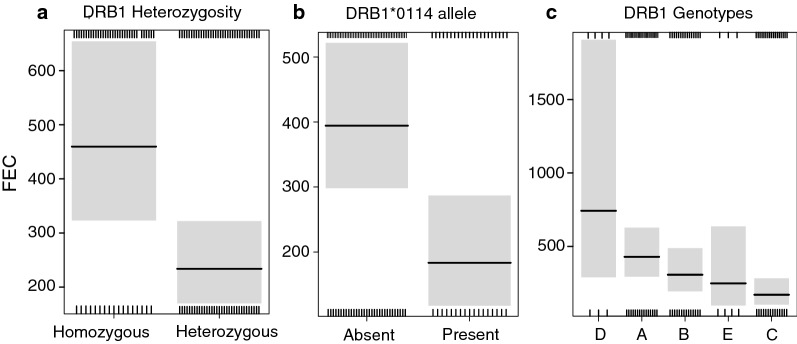
Predicted GINs burden (FEC) values obtained from best genetic models for (**a**) DRB1 heterozygous and homozygous individuals, (**b**) for individuals carrying or not carrying the DRB1*0114 allele or (**c**) individuals carrying one of the DRB1 genotype. Black lines represent predicted values and grey bands represent the 95% confidence interval. Upper and lower ticks represent the number of positive and negative residuals, respectively

Almost all GINs models including genetic predictors (16 out of 19) had a lower AICc than the non-genetic model, highlighting the strong relationship between GINs resistance and genetics. In particular, the model including DRB1 heterozygosity (model set (i)) was the best model (lowest AICc), indicating that among the three DRB1 characteristics evaluated (heterozygosity, alleles and genotypes), heterozygosity was the best descriptor of parasite resistance for GINs. The model including both sMLH/sMLH^2^ and DRB1 heterozygosity was better than the models including only DRB1 heterozygosity or sMLH/sMLH^2^ (∆AICc > 2, Table 2). A significant difference of 52% in averaged FEC was detected between heterozygous and homozygous individuals (Fig. 2a). When testing the effects of specific alleles at DRB1 locus on FEC (model set (ii)), the best model was the model including sMLH/sMLH^2^ and DRB1*0114 allele (Table 2). Estimate was negative for the presence of this allele (Table 3) and its presence led to a 56% decrease in FEC between individuals carrying or not carrying this allele (Fig. 2b). Finally, in the model set (iii), the models including sMLH/sMLH^2^ and DRB1 genotypes or only DRB1 genotypes were better than the non-genetic model (∆AICc > 2, Table 2). We found a marked gradient (Fig. 2c) between the most parasitized DRB1 genotype (D) and the least parasitized genotype (C) with a statistically significant difference between A and C genotypes, leading to a 57.2% decrease in averaged FEC. The *F*-ratio test between “local” and “global” models revealed no significant differences, indicating stronger support for the global hypothesis (*F* = 0.96, *d.f.* = 37, *p* = 0.54).

## Discussion

As illustrated here, parasite resistance in the female Mediterranean mouflon is a complex trait controlled by several non-genetic and genetic predictors. For both parasite classes, individuals in better condition were less parasitized. Multi-locus heterozygosity was linked to GINs resistance through a U-shaped relationship suggesting the presence of both in- and outbreeding depression in our population. However, since *g*_*2*_ and the “global/local” test did not lead to same conclusions, we were not able to distinguish between local and global effects of neutral genetic variation. It seemed that DRB1 candidate locus conferred a heterozygote advantage and that rare alleles and/or fluctuating selection might also occur in the study population [90]. These results confirm that the three main hypotheses about HFCs are not mutually exclusive [91]. In contrast, while coccidia burden appeared as simultaneously driven by age, day of sampling and time lapse between sampling and coproscopy, we detected no genetic predictor effects for that class of parasites, illustrating that resistances to different parasite classes (here GINs and coccidia) are driven by different characteristics (see also [85, 86]), emphasizing the importance of performing multi-specific studies.

### Different characteristics are determining different parasite resistances

None of the genetic predictors studied were linked with coccidia resistance. The absence of correlation between genetic diversity and parasite resistance was also observed in other host-parasite systems (e.g., [41, 92, 93]). Although a lack of statistical power cannot be excluded to explain this result, the genetic effects detected for GINs with the same dataset suggested that genetics had much less effect on variation in micro-parasite resistance than in macro-parasite resistance. Repeatability was notably lower for FOC than FEC (yet comparable to other studies, e.g., [94]), indicating that variation in FOC is primarily driven by short-term effects or measurement errors, rather than genetic effects.

Differences between results for coccidia and GINs may be due to the fact that coccidia are intracellular protozoa, while GINs are macro-parasitic nematodes. Micro- and macro-parasites are thought to be controlled by different immune responses (Th1 and Th2 respectively [85, 86]) that can be involved in trade-offs and thus not active at the same time (e.g., [86, 87], see also [88] for a review). Different immune pathways may be impacted by different genetic factors explaining the differences observed between GINs and coccidia in the present study. MHC class II genes such as DRB1 seem also more specifically linked to an extracellular parasite-derived peptide presentation ([80, 95]) that may explain the impacts of DRB1 on GINs but not on coccidia.

### Neutral genetic diversity effects on nematode resistance

We observed a U-shaped relationship between sMLH and GINs burden with a maximal parasite resistance obtained for individuals with intermediate heterozygosity levels. Parasite burden decreased with increasing heterozygosity until a threshold (~ 1), after which highly heterozygous individuals were parasitized as much as highly homozygous individuals, suggesting the presence of both positive and negative HFCs. While a positive relationship between parasite resistance and genetic diversity is the rule (e.g., [34, 35, 45, 79, 96]), quadratic relationships have also been previously reported (e.g., in Soay sheep [36]; lesser kestrel, *Falco naumanni* [56]; rostrum dace, *Leuciscus leuciscus* [97]; raccoons [33]; blue tits, *Cyanistes caeruleus* [98]) but most often in the opposite direction with individuals carrying intermediate heterozygosity levels being less resistant (see e.g., [33, 97, 98]). Optimal parasite resistance was nevertheless observed for an intermediate level of genetic diversity in studies considering the number of MHC alleles [61, 99]. Indeed, when considering encoding genes such as MHC genes, theory predicts that while a high diversity of alleles enables a large spectrum of pathogen recognition (diversifying selection), it could also limit the immune response efficiency by causing self-reacting [100]. Accordingly, an intermediate number of alleles is expected to confer the highest fitness to individuals due to the two contradictory evolutionary forces acting on MHC diversity. The U-shaped relationship observed here for multi-locus heterozygosity might thus suggest that two contradictory evolutionary forces are also acting on neutral genetic diversity.

A positive relationship between genetic diversity and fitness-related traits such as parasite resistance can be explained by inbreeding depression with more inbred individuals exhibiting lower levels of heterozygosity and fitness [101]. On the other hand, negative HFCs and thus heterozygote disadvantage might be explained by outbreeding depression (i.e. reduced fitness in offspring originating from highly differentiated parents) [102]. Negative HFCs have been documented much less than positive ones [103–105] (but see e.g., [45, 106, 107]) but the U-shaped relationship observed here may suggest the presence of both inbreeding and outbreeding depression in our population. In- and outbreeding depression co-occurrence have been observed within the same populations (e.g., [108, 109]) and on the same fitness traits [103, 110–112]. It requires that population structure (e.g. philopatry, founder events) induce both local adaptation and inbreeding in the population [111]. Due to high female philopatry in the study population [113, 114], moderate inbreeding (a low number of individuals exhibiting low sMLH) is likely to occur in females. On the other hand, the release of founders originating from three diverse origins [115] is likely to have generated outbreeding depression that still persists as observed in this population for other genetic signals [114]. Outbreeding depression might result from underdominance, disruption of epistatic interactions leading to break-down of co-adapted gene complexes and/or loss of local adaptations by disruption of advantageous gene × environment interactions [102].

Finally, the absence of support for the local effect hypothesis suggested that the observed HFC was due to a genome-wide diversity effect. However, *g*_*2*_ was not significantly different from zero, preventing us from coming to a conclusion about global or local effect of multi-locus heterozygosity. Detection of significant identity disequilibrium using *g*_*2*_ is only rarely achieved (see [116]) and numerous studies have evidenced significant HFCs despite no detectable identity disequilibrium [34, 52, 116, 117]. Accordingly, studies where *g*_*2*_ and global/local tests [52] highlighted opposing results are not scarce (see e.g., [91, 118]). However, even when not detected, local effects cannot be fully discarded since their detection is very difficult due to dilution effects of unlinked loci on linked loci (see [52] but see e.g., [91]).

### Candidate gene effects on nematode resistance

Links between MHC heterozygosity and fitness were evidenced across a wide range of taxa (e.g., [38, 39] but see [41]). Three main mechanisms that can co-occur have been proposed to explain the impacts of MHC diversity on pathogen resistance: (i) heterozygote advantages (i.e. heterozygote recognizing and binding a wider range of antigens than homozygotes, through overdominance or dominance), (ii) rare-allele advantages (negative frequency-dependence) in which new alleles confer advantages since selection favors parasites overcoming the more common resistance alleles and (iii) fluctuating selection proposing that spatio-temporal variability of pathogen types and abundances induce fluctuating selection on MHC, inducing differential links between pathogen resistance and MHC diversity (see [90] for a review). Heterozygous advantage can be detected when MHC heterozygosity and parasite resistance are associated, while rare-allele and fluctuating selection will be detected through specific MHC allele effects on resistance [90]. In the present study, genotypes, specific alleles and heterozygosity effects were evidenced suggesting that heterozygote advantage, rare-allele effects (e.g. DRB1*0114 allele was the rarest) and/or fluctuating selection might occur. Genotypes effects were the weakest and seemed mostly linked to heterozygosity effects. Indeed, although differences between genotypes were not significant, heterozygous genotypes (genotypes B, C, and E) were significantly less parasitized than homozygous genotypes (see Fig. 2a, c). Heterozygosity effects were also stronger than specific allele effects, supporting a predominant heterozygote advantage. Distinguishing between the overdominance and dominance explanation for heterozygote advantage is challenging [90], but heterozygous individuals were less parasitized than both types of homozygous individuals, suggesting that the heterozygote advantage we observed was due to overdominance (see [38, 90]). Evaluating impacts of parasitism on survival and/or reproductive success might help to determine through which trait heterozygote advantage occurs.

Specific allele effects might also be explained in the light of heterozygosity effects. Indeed, the negative effects of DRB1*0114 on FEC could be attributed to the fact that it was only present in heterozygous individuals (genotype F individuals removed from the dataset because of a too-small sample size). However, models containing alleles were among the best models, and specific allele effects might more likely be due to the immunological properties of their products (i.e. peptide binding sites in our case). Specific MHC and DRB1 allele effects on fitness and parasite resistance were observed elsewhere (e.g., [41, 81, 119]). However, to our knowledge, the three DRB1 alleles sequences identified in our population were previously observed in only one study [89]. Hermann-Hoesing et al. [89] studied the impacts of DRB1 alleles on ovine progressive pneumonia virus resistance in domestic ewes. They evidenced that allele DRB1*0324 and DRB1*0114 were associated with a higher provirus level, while DRB1*07012 allele was associated with a lower provirus level. The authors explained that these differences were linked with specific amino-acid encoded by the diverse alleles and determining the immune response. Indeed, immunological theory predicts that specific alleles could be advantageous (disadvantageous) if their products are more (less) effective in presenting pathogen-derived peptides [120]. Thus our results suggested that protein binding sites encoded by DRB1*0114 conferred an advantage against GINs infections. Since different functional links between genetics and resistance could indeed be expected when considering different parasite classes (see e.g., [33, 48, 56, 57]), the opposing effects of DRB1*0114 observed between Herrmann-Hoesing et al. [89] and the present study are not surprising since provirus and macro-parasitic strongyles are very different pathogen types.

## Conclusions

Our findings brought important insights for Mediterranean mouflon and more generally for large ungulate management. Firstly, the positive impact of genetic diversity on parasite resistance detected emphasizes the importance of promoting genetic diversity and preventing inbreeding in populations. Gene flow [28, 121–124] and thus genetic diversity (e.g., [125, 126]) might be impacted by landscape in wild sheep and ungulates. Accordingly, careful attention must be given to maintaining landscape connectivity, especially in threatened populations (e.g. Corsican mouflon [73], Argali, *Ovis ammon* [75, 127, 128], Cypriot mouflon, *Ovis orientalis ophion* [74], Sierra Nevada bighorn sheep, *Ovis canadensis sierra* [129, 130]). Secondly, when planning introductions or translocations in conservation and genetic reinforcement strategies, maximizing the admixture of founder/translocated individuals might increase parasite resistance by increasing genetic diversity [131]. Similarly, in accordance with the direct effects of the DRB1 gene, translocated individuals might be chosen according to their parasite resistance characteristics (e.g. carrying resistance alleles). We nevertheless also evidenced that outbreeding depression can decrease parasite resistance. Wildlife managers must thus be careful regarding local adaptations when choosing individuals and source populations. In addition, in wild populations, another concern when introducing new individuals might be the introduction of alien parasite species which might have substantial negative consequences [132–134]. Finally, gathering more data on males would allow us to determine if genetic effects are sex-specific, and to measure the impacts of selective hunting on parasite resistance. Indeed, parasite-mediated sexual selection [135] posits that secondary sexual characteristics, such as horns, are an honest signal about parasitism rates of males (see e.g., [34, 136] but see [137]). Since in most wild sheep and Mediterranean mouflon populations males are hunted for their trophies, hunting can counter natural selection and could modify resistance allele frequencies [59, 138–140].

## Methods

### Study population and data collection

The Mediterranean mouflon study population originates from the release of 19 individuals between 1956 and 1960 [115] in a National Hunting and Wildlife Reserve (1658 ha, 532–1124 m above sea level; hereafter called “reserve”) in the Caroux-Espinouse massif (43°38′N, 2°58′E, 17,000 ha, 130–1124 m asl, southern France). Vegetation is composed of beech, chestnut and coniferous forests in this low mountain area where deep valleys and plateaus draw a mosaic of ridges and talwegs (i.e. lines of lowest elevation within a valley, see [141, 142] for details). Local climate is under the influence of Mediterranean, oceanic and mountainous weather patterns [143] with dry and hot summers, autumns with lots of precipitation and cold winters [144].

The population has been monitored each year since 1974, mainly during spring and early summer (April–July), by capture-mark-recapture. Animals were baited with salt and captured using individual or collective traps and dropping nets. When captured, animals were marked with a numbered/colored collar; biometric measurements were made and hairs and faeces were sampled for genetic and coproscopic analyses. Genetic analyses revealed that gene flow is mostly insured by male reproductive dispersal (reproductive excursions, [114]), while ewes are philopatric [113, 145, 146]. Females exhibit a significant socio-spatial genetic structure consisting primarily of three spatially disconnected and genetically differentiated units (*Nf*, *Cf* and *Sf*, see [114]) and gene flow has been shown to be impacted by several landscape features [124].

### Genetic analyses

#### Neutral genetic diversity

Individuals were genotyped at 16 microsatellite markers (see [114] for details) using hair samples. Genotyping was performed by the Antagene laboratory (Limonest, France, www.antagene.com) following the procedure presented in Portanier et al. [114]. To assess if genome-wide genetic diversity was associated with parasite resistance, we calculated the standardized multi-locus heterozygosity (sMLH) for individuals having at least 13 microsatellite markers. sMLH was calculated as the ratio between the proportion of loci at which an individual was heterozygous and the mean heterozygosity of typed loci (see [36]) using the *inbreedR* package for R software [147]. To determine if our set of markers was a good proxy for genome-wide heterozygosity and discriminate between global and local effects of sMLH, we quantified identity disequilibrium in the whole population and within each socio-spatial unit by estimating *g*_2_, a measure of the covariance in heterozygosity using Robust Multi-locus Estimates of Selfing software (RMES [148]) with 10,000 iterations. RMES tests whether *g*_*2*_ is significantly different from zero. If *g*_*2*_ = 0, HFCs are not expected to appear because identity disequilibrium is not expected to be present in the population.

#### Candidate gene approach

The second exon of the MHC-DRB class II gene encoding the ligand-binding domain of the protein was amplified and sequenced for all individuals. Each sample was analyzed twice by at least two independent technical replicates. Briefly, we performed the two-step PCR strategy combined with the dual-index paired-end sequencing approach described in Galan et al. [149]. During the first PCR, we used a modified version of the primers LA31 (5′-GATCCTCTCTCTGCAGCACATTTCCT-3′) and LA32 (5′-TTCGCGTCACCTCGCCGCTG-3′) initially designed for cattle [150], with the addition of a partial overhang Illumina sequencing primers in 5′-end. The first PCRs were carried out in a 10 µL reaction volume using 5 µL of Multiplex PCR Kit (Qiagen) and 0.5 mM of each primer. We added to each well a volume of 1.5 µL of DNA. This PCR consists of an initial denaturation at 95 °C for 15 min, followed by 40 cycles of denaturation at 94 °C for 30 s, annealing at 55 °C for 30 s and extension at 72 °C for 30 s, with a final extension phase at 72 °C for 10 min. The second PCR consists of a limited-cycle amplification step to add multiplexing indices i5 and i7 and Illumina sequencing adapters P5 and P7 at both ends of each DNA fragment (see [149] for details). The PCR products were verified by electrophoresis in a 1.5% agarose gel. One negative control for extraction, one PCR blank and one negative control for indexing were systematically added to each of the PCR microplates. Each DNA extraction was amplified and indexed in two independent PCR reactions. These PCR replicates were used as technical replicates to confirm the genotypes and further remove the false-positive results [151]. PCR products were pooled by volume and a 2 × 250 bp paired-end MiSeq (Illumina) run was conducted. The SESAME barcode software (SEquence Sorter & AMplicon Explorer [152]) was used to sort sequences, identify and discard artefactual variants, and generate the haplotypes and individual genotypes.

In the candidate gene approach, we also genotyped a microsatellite located in the gamma interferon gene (chromosome 3, o(IFN)-γ) known to be linked with parasite resistance in wild sheep (see e.g., [59]). However, based on the analysis of a representative subset of 48 individuals, we found the o(IFN)-γ to be monomorphic in our population and it was thus not considered in subsequent analyses (data not shown).

### Fecal parasite egg and oocyst counts

Mediterranean mouflon might be infected by a large diversity of endoparasites such as *Trichuris* spp., *Moniezia* spp. or *Dicrocoelium* spp. but the most prevalent are strongylid nematodes and coccidia (*Eimeria* spp., [30, 153]). We accordingly limited our analyses to these last two parasites types. Strongyles and coccidia abundances were estimated by counting the number of eggs and oocysts in fecal samples (FEC and FOC, respectively, are widespread parasite resistance measurements often used in HFCs studies; see e.g., [3, 34]). FEC and FOC represented the abundances of all strongyles and coccidia species present in the samples, respectively. Coproscopic analyses were performed between 2010 and 2017. Faeces samples were individually stored in a refrigerated container before analyses. FEC and FOC were estimated using a modified MacMaster procedure (modified from [154]). After sample homogenization, 5 grams of faeces were weighed and mixed with 70 mL of zinc sulfate (d = 1.36). The sample was then filtered through a sieve lined with a compress and the homogenized filtrate was immediately loaded in two 0.15 mL chambers of a MacMaster slide. After allowing them to float at the surface for at least one minute, eggs and oocysts were counted using a compound microscope (magnification ×100). The number of eggs or oocysts per gram of faeces (EPG and OPG, respectively) was obtained by multiplying the total number of counted eggs by 50. In order to perform a qualitative examination (“control slide” hereafter), we filled a 14 mL tube with the remaining solution until a meniscus was obtained; a cover slide was then placed on the tube. After 5 min of centrifugation at 1200 rpm, the cover slide was recovered and placed on a microscope slide. We searched for parasite propagules using a microscope (magnification ×40–400). The theoretical sensitivity of the MacMaster is 50 EPG/OPG of fecal matter. When, for an individual, no eggs or oocysts were observed using the MacMaster technique, but at least one egg or oocyst was observed on the control slide, we attributed the value of 25 EPG/OPG for FEC or FOC. FEC and FOC had skewed distribution and were log-transformed to obtain a normal distribution. To avoid log of zero for GINs, 10 was added to FEC values. We assessed repeatability of FEC and FOC measurements for a given animal by computing intra-class correlation coefficients (for unbalanced design because number of measurements differed among animals [155]).

### Statistical analyses

Prior to testing for genetic effects on FEC and FOC we first identified (and accounted for) other intrinsic and extrinsic variables known to impact parasitism [18, 20, 22, 78, 156–158]. Variables considered included the year and the Julian date of sampling to account for intra and inter-annual variations in environmental conditions and population densities. We corrected for part of the sampling variance by adding the number of days between sampling and coproscopic analyses in the models since it can impact the number of fecal egg and oocyst counted [159, 160]. We only considered individuals for which less than 30 days elapsed between the sampling and laboratory analyses. Body condition (Scaled Mass Index, calculated based on individual mass and metatarsus length [161]) was included to account for heterogeneity in individual quality, and age was included to account for changes in immunity with age (e.g., [22, 162–164]). Since females cannot be accurately aged when > 3 years old [165], ages were divided into 4 classes: 1, 2, 3 and ≥ 4-year-old individuals. However, due to the paucity of data on males and juveniles, we focused in this study on sexually mature females only (i.e. 2 or more years old [115]). A total of 79 individuals representing 120 observations were included in subsequent analyses. Finally, the socio-spatial unit (SSU) of individuals was also included (80, 28 and 10 observations from the *Cf*, *Nf* and *Sf* socio-spatial units, respectively) since spatial structure of the population, when overlooked, can lead to spurious HFCs [166].

We applied a two-stage procedure in a linear mixed model selection framework, first identifying for each response variable the best non-genetic model (including both extrinsic factors and other, not purely molecular, intrinsic factors) to which genetic predictors (both neutral and adaptive) were then added. The predictors included in non-genetic models can also have a genetic basis (e.g., body condition [167]) but are different from direct genetic measurements such as the ones included in genetic models. Such two-stage procedure enabled measurement of the importance of genetic predictors relative to other factors known to impact parasite resistance, and to avoid over-parameterization of models. Model selection was done by comparing corrected Akaike’s Information Criterion (AICc) values of the possible models. All variables included in models being in a range of ∆AICc < 2 were included in the optimal non-genetic model. We then evaluated the improvement of models through the addition of genetic predictors. Since there could be specific and/or general effects of genetic diversity on FEC and FOC, three sets of genetic models were created and included (i) sMLH and/or the DRB1 heterozygosity status of individuals coded as 1 if heterozygous and 0 if homozygous, (ii) sMLH and/or the presence of specific DRB1 alleles, coded as 1 if present and 0 if absent (see e.g., [41] for a similar approach on survival), and (iii) sMLH and/or the DRB1 genotypes of individuals.

All continuous variables (sMLH, day of sampling, body condition, time elapsed between sampling and coproscopy) were centered and scaled (mean = 0, standard deviation = 1) before analyses and individual identity and year of sampling were included as random effects to account for repeated measurements and measurements made in different years, respectively. We also tested for possible non-linear relationships between FEC and FOC and continuous variables by adding their quadratic terms in models. If the addition of the quadratic term did not improve the model by more than two AICc units (ΔAICc > 2), only the linear term was retained to perform model selection. Multi-collinearity of predictors was checked using variance inflation factors (VIF, *vif.mer* function [168]). Following Zuur et al. [169], if a predictor showed a VIF > 3, it was not kept in model selection. In both non-genetic and genetic steps, residuals structure and normality of the best models were tested and visually assessed (see Additional file 2). We measured the relative likelihood of each model using the AIC weights. Goodness-of-fit was assessed using conditional (R^2^c) and marginal (R^2^m) R^2^, representing the variance explained by the fixed and random effects and by the fixed effects alone, respectively.

Finally, in order to discriminate between the hypotheses of local or global effects of neutral genetic diversity on parasite resistance, we followed the method proposed by Szulkin et al. [52]. We built two models including the non-genetic predictors retained and (i) sMLH, and its quadratic term if necessary, as the sole genetic predictor (“global” model) or (ii) all single locus heterozygosities, with each individual coded as 1 if heterozygous or 0 if homozygous (“local” model). The two models were then compared using a *F*-ratio test. If the “local” model explains more variance than the “global” model, the local effect hypothesis will receive more support than the global one. Since there are relatively large differences in loci heterozygosity levels (see [114]), we also performed the test using the standardized approach introduced by Szulkin et al. [52], in which more weight is given to more heterozygous loci. Both non-standardized and standardized approaches led to the same results; only results of the standardized approach are given in the following. All modeling and model selection were performed using *lme4* [170] and *MuMIn* packages [171] of R 3.3.1 software [172]. Significance tests were performed using the *lmerTest* package [173] and model plots were carried out using the *visreg* package [174] of R 3.3.1 software [172]. The code used for all the statistical analyses is available in the Additional file 4.

## Additional files

**Additional file 1.** Results of non-genetic model selection.

**Additional file 2.** Normality tests of non-genetic and genetic models.

**Additional file 3.** Variance Inflation Factors values for genetic models and tests of the presence of sMLH quadratic effects on FOC and FEC.

**Additional file 4.** R code used for statistical analyses.
