# Supplementary material for: Both candidate gene and neutral genetic diversity correlate with parasite resistance in female Mediterranean mouflon
Source: BMC Ecol. 2019 Mar 5;19:12. doi: 10.1186/s12898-019-0228-x (PMC6402107; doi:10.1186/s12898-019-0228-x)

***Both candidate gene and neutral genetic diversity correlate with parasite resistance in female Mediterranean mouflon***

Elodie Portanier^1, 2, 3^, Mathieu Garel^2^, Sébastien Devillard^1^, Daniel Maillard^2^, Jocelyn Poissant^4^, Maxime Galan^5^, Slimania Benabed^3^, Marie-Thérèse Poirel^3^, Jeanne Duhayer^2^, Christian Itty^2^ and Gilles Bourgoin^1, 3^

*^1^Univ Lyon, Université Claude Bernard Lyon 1, CNRS, Laboratoire de Biométrie et Biologie Évolutive, F-69100, Villeurbanne, France.*

^2^*Office National de la Chasse et de la Faune Sauvage, Unité Ongulés Sauvages, 5 allée de Bethléem, Z.I. Mayencin F-38610, Gières, France.*

^3^*Université de Lyon, VetAgro Sup, Campus Vétérinaire de Lyon, 1 Avenue Bourgelat, BP 83 F-69280, Marcy l’Etoile, France.*

*^4^Department of Ecosystem and Public Health, University of Calgary, Calgary, Canada.*

*^5^CBGP, INRA, CIRAD, IRD, Montpellier SupAgro, Université de Montpellier, F-34980, Montferrier sur Lez, France.*

**Correspondence:** Elodie Portanier, Université Claude Bernard Lyon 1, CNRS, Laboratoire de Biométrie et Biologie Évolutive, 69100, Villeurbanne, France, Fax: +33 4 72 43 13 88, E-mail: elodie.portanier@gmail.com

Additional file 1

Results of non-genetic model selection

None of the non-genetic predictors showed VIF higher than three indicating no correlations issues (Table S1). No quadratic term of non-genetic predictors improved AICc values by more than two indicating no quadratic relationship between FEC and FOC and ICC, the time elapsed between sampling and coproscopy and the Julian date of sampling (Table S2). Regarding FOC, the best model (lowest AICc) included age-classes and the time lapse between sampling and coproscopy (Table S3). However, the six first models were equivalent (ΔAICc < 2) and also included the day of sampling and the SMI as significant variables (Table S3). The best non genetic model retained for *Eimeria* spp. thus accounted for these four non-genetic variables and had a goodness-of-fit of R²c = 0.34, R²m = 0.15. The averaged estimates of FOC for age-classes 2 and 3 were β = 1.39 ± 0.39 and 0.71± 0.28, respectively and were significantly different of those of individuals from age-class > 4 (intercept, *p* < 0.05). Parasite burden decreased with age-classes (Figure S1). The estimate for the time lapse between sampling and coproscopy was β = 0.07 ± 0.11 (*p* > 0.05) indicating that parasite burden linearly increased with the number of days elapsed between sampling and coproscopic analyses (Figure S1). On the opposite, the relationship between FOC and SMI or the Julian day of sampling was negative (β = -0.03 ± 0.07 and -0.02 ± 0.06, respectively, *p* > 0.05, Figure S1) indicating that individuals in better body condition were less parasitized and that parasite burden decreased linearly during the capture period.

Regarding FEC, the best model included only effects of SMI (Table S3) and all subsequent models had a ΔAICc > 2. The best non-genetic model retained for GINs thus only accounted for SMI and had a goodness-of-fit of R²c = 0.45, R²m = 0.15. Relationship between FEC and SMI was negative (β = -0.53 ± 0.12, *p* < 0.001, Figure S2) indicating that individuals in better condition had lower GINs burden.

Table S1: Variables used in non-genetic mixed-effects linear models and relative Variance Inflation Factors values (VIF) for both FEC and FOC. SMI stands for the body condition, SSU is the socio-spatial unit of individuals, age-class is the age class of individuals, time lapse represent the time elapsed between sampling and coproscopic analyses and Julian day is the Julian day of sampling. All models included the individual identity and the year of sampling as random effects.

|  | SMI | SSU *Nf* | SSU *Sf* | Age-class 2 | Age-class 3 | Time lapse | Julian day |
| --- | --- | --- | --- | --- | --- | --- | --- |
| FEC | 1.22 | 1.14 | 1.28 | 1.11 | 1.06 | 1.50 | 1.43 |
| FOC | 1.23 | 1.12 | 1.26 | 1.12 | 1.07 | 1.43 | 1.38 |

Table S2: Model selection of mixed-effects models based on corrected Akaike’s Information Criterion (AICc) for testing the effects of quadratic terms of continuous non-genetic predictors on parasite resistance as measured by FOC and FEC. SMI stands for the body condition, SSU is the socio-spatial unit of individuals, age is the age class of individuals, Julian day is the Julian day of sampling and time lapse represent the time elapsed between sampling and coproscopic analyses. All models included the individual identity and the year of sampling as random effects.

| Response variable | Component of the model | AICc |
| --- | --- | --- |
| FOC | SMI + Julian day + time lapse + SSU + age | 382.97 |
|  | SMI + SMI² + Julian day + time lapse + SSU + age | 384.43 |
|  | SMI + Julian day + Julian day² + time lapse + SSU + age | 385.37 |
|  | SMI + Julian day + time lapse + time lapse² + SSU + age | 384.57 |
| FEC | SMI + Julian day + time lapse + SSU + age | 398.40 |
|  | SMI + SMI² + Julian day + time lapse + SSU + age | 400.37 |
|  | SMI + Julian day + Julian day² + time lapse + SSU + age | 399.81 |
|  | SMI + Julian day + time lapse + time lapse² + SSU + age | 399.48 |

Table S3: Model selection of mixed-effects models based on corrected Akaike’s Information Criterion (AICc) for testing the effects of non-genetic predictors on parasite resistance as measured by FOC and FEC. D.f are the degree of freedom, weight is the Akaike weight. Only the best models are shown. SMI stands for the body condition, SSU is the socio-spatial unit of individuals, age is the age class of individuals, Julian day is the Julian day of sampling and time lapse represent the time elapsed between sampling and coproscopic analyses. All models included the individual identity and the year of sampling as random effects.

|  | d.f. | Log-likelihood | AICc | ΔAICc | Weight |
| --- | --- | --- | --- | --- | --- |
| **FOC** |  |  |  |  |  |
| age + time lapse | 7 | -180.10 | 375.2 | 0.00 | 0.23 |
| age | 6 | -181.36 | 375.5 | 0.26 | 0.20 |
| age + Julian day | 7 | -180.86 | 376.7 | 1.53 | 0.11 |
| age + time lapse + SMI | 8 | -179.73 | 376.8 | 1.57 | 0.10 |
| age + SMI | 7 | -181.01 | 377.0 | 1.82 | 0.09 |
| age + time lapse + Julian day | 8 | -180.08 | 377.5 | 2.26 | 0.07 |
| age + SMI + Julian day | 8 | -180.54 | 378.4 | 3.19 | 0.05 |
| **FEC** |  |  |  |  |  |
| SMI | 5 | -188.54 | 387.6 | 0.00 | 0.39 |
| SMI + Julian day | 6 | -188.52 | 389.8 | 2.19 | 0.13 |
| SMI + time lapse | 6 | -188.54 | 389.8 | 2.22 | 0.13 |
| SMI + age | 7 | -187.79 | 390.6 | 2.99 | 0.09 |
| SMI + SSU | 7 | -187.91 | 390.8 | 3.23 | 0.08 |

Figure S1: Predicted *Eimeria* spp. burden (FOC) values a function of age-classes (A), scaled time lapse (scaled number of days) between sampling and coproscopic analyses (B), SMI (C) and scaled Julian day of sampling (D) from the best non genetic model. Individuals of 2 (3) years are grouped in the class “2” (“3”) while class “>4” grouped individuals 4 and more years old. Black lines represent predicted values and grey bands represent the 95% confidence interval. Upper and lower ticks represent positive and negative residuals, respectively.


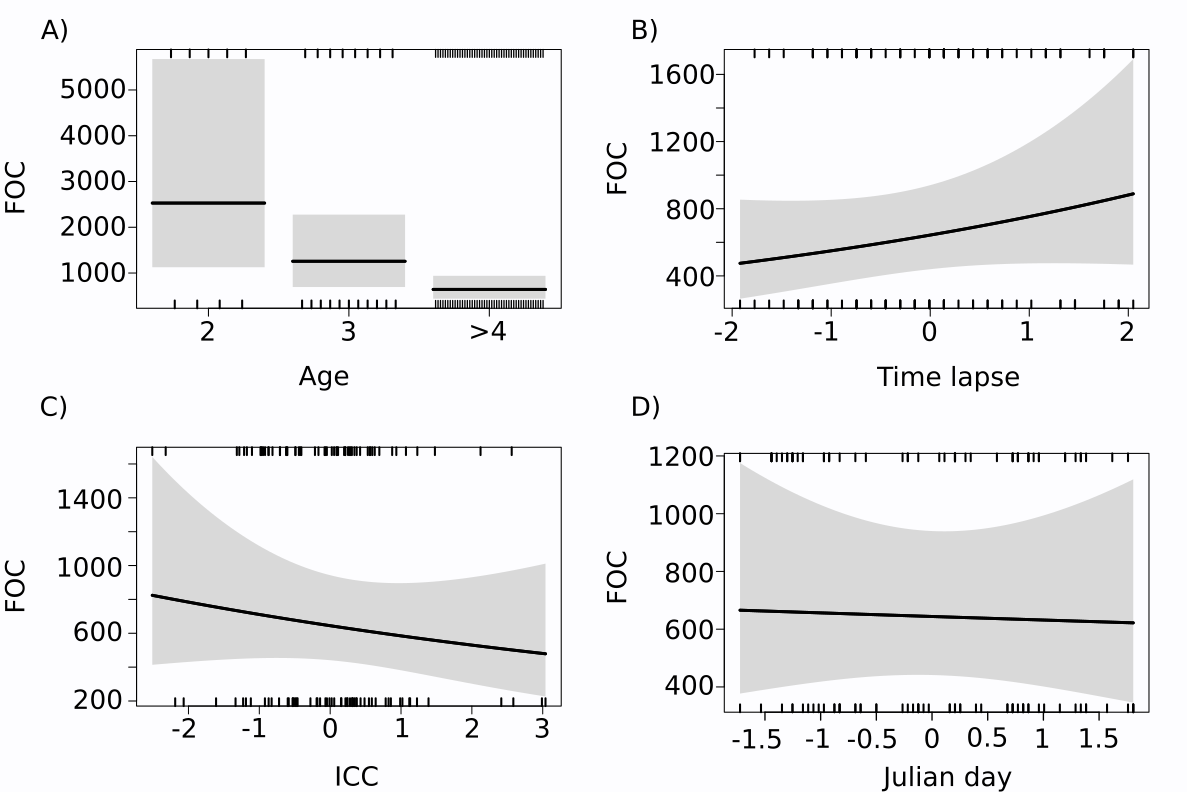


Figure S2: Predicted GINs burden (FEC) values a function of scaled SMI from the best non genetic model. Black lines represent predicted values and grey bands represent the 95% confidence interval. Upper and lower ticks represent positive and negative residuals, respectively.


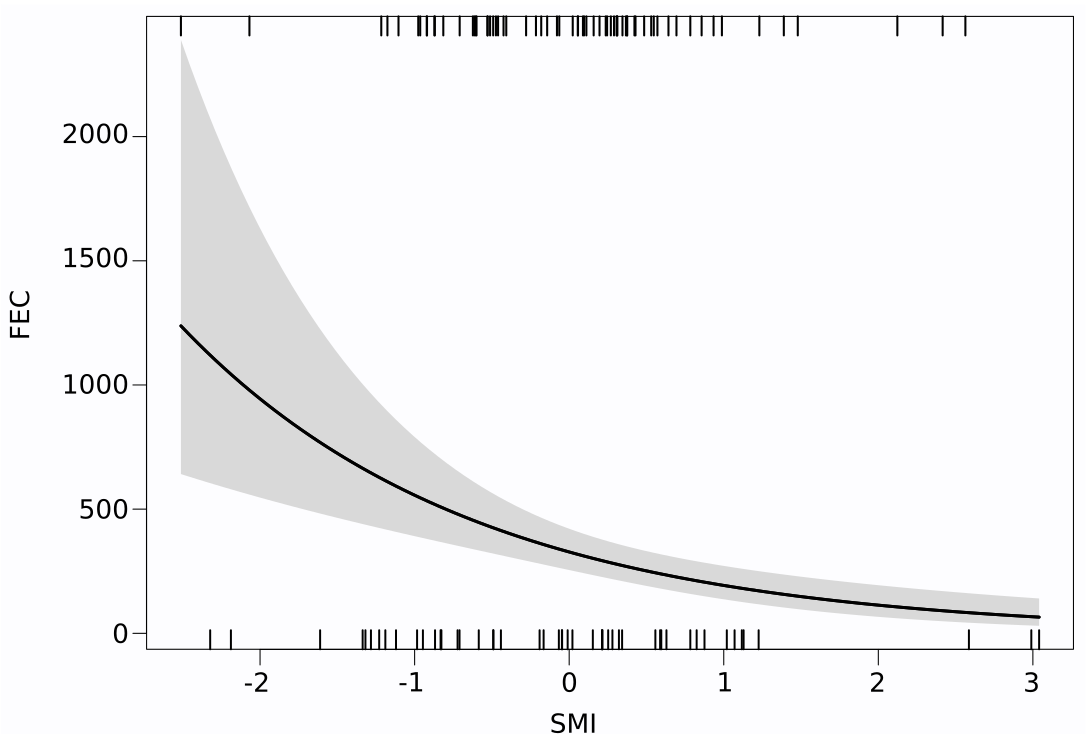

Supplement: Supplementary file 1 — Additional file 1. Results of non-genetic model selection. [file 12898_2019_228_MOESM1_ESM.docx]
