# Supplementary material for: Both candidate gene and neutral genetic diversity correlate with parasite resistance in female Mediterranean mouflon
Source: BMC Ecol. 2019 Mar 5;19:12. doi: 10.1186/s12898-019-0228-x (PMC6402107; doi:10.1186/s12898-019-0228-x)

***Both candidate gene and neutral genetic diversity correlate with parasite resistance in female Mediterranean mouflon***

Elodie Portanier^1, 2, 3^, Mathieu Garel^2^, Sébastien Devillard^1^, Daniel Maillard^2^, Jocelyn Poissant^4^, Maxime Galan^5^, Slimania Benabed^3^, Marie-Thérèse Poirel^3^, Jeanne Duhayer^2^, Christian Itty^2^ and Gilles Bourgoin^1, 3^

*^1^Univ Lyon, Université Claude Bernard Lyon 1, CNRS, Laboratoire de Biométrie et Biologie Évolutive, F-69100, Villeurbanne, France.*

^2^*Office National de la Chasse et de la Faune Sauvage, Unité Ongulés Sauvages, 5 allée de Bethléem, Z.I. Mayencin F-38610, Gières, France.*

^3^*Université de Lyon, VetAgro Sup, Campus Vétérinaire de Lyon, 1 Avenue Bourgelat, BP 83 F-69280, Marcy l’Etoile, France.*

*^4^Department of Ecosystem and Public Health, University of Calgary, Calgary, Canada.*

*^5^CBGP, INRA, CIRAD, IRD, Montpellier SupAgro, Université de Montpellier, F-34980, Montferrier sur Lez, France.*

**Correspondence:** Elodie Portanier, Université Claude Bernard Lyon 1, CNRS, Laboratoire de Biométrie et Biologie Évolutive, 69100, Villeurbanne, France, Fax: +33 4 72 43 13 88, E-mail: elodie.portanier@gmail.com

Additional file 2

Normality tests of (non)-genetic models

Residuals of the non-genetic model for *Eimeria* spp. followed a normal distribution (Shapiro-Wilk W = 0.99, *p* = 0.82) while not for GINs (Shapiro-Wilk W = 0.98, *p* = 0.03). The significance of the Shapiro-Wilk normality tests for GINs was nevertheless though to be due to only few points (see Figure S3) and since p-value was closed to 0.05, normality could be assumed. In addition, none of the best non-genetic models had structured residuals (Figure S4).

When adding the genetic predictors, for FEC, the full genetic models (ii) and (iii) had normal residuals (W = 0.98, *p* = 0.07 and W = 0.98, *p* = 0.08, respectively). Regarding model set (i), residuals of the full genetic model did not follow a normal distribution (W = 0.98, *p* = 0.03). However, as previously, the significance of the Shapiro-Wilk normality tests for GINs was thought to be due to only few points (see Figure S5) and since *p-value* was closed to 0.05, normality could be assumed. In addition, one of the best genetic models had structured residuals (Figure S6).

Figure S3: QQplot of GINs in non-genetic model


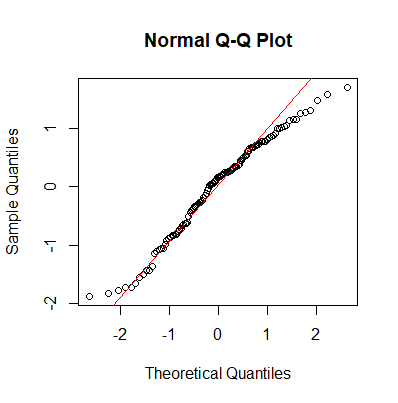


Figure S4: Residuals of best non-genetic models for Eimeria spp. (A) and GINs (B) plotted against fitted values.


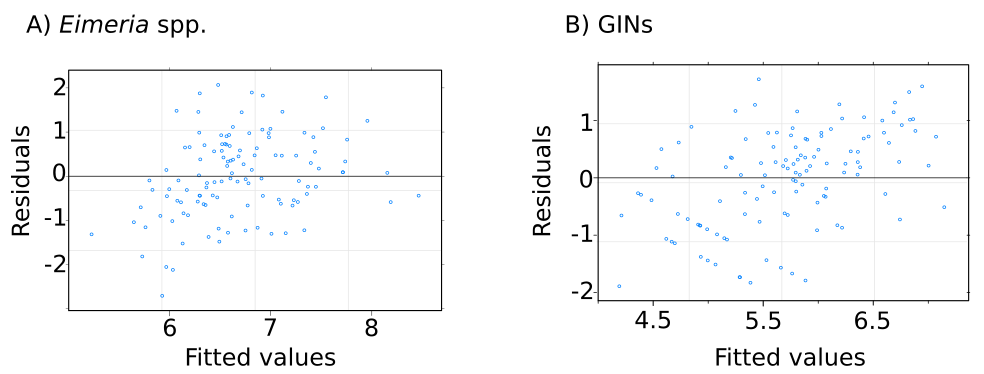


Figure S5: QQplot of GINs in genetic model set (i) testing the effects of DRB1 heterozygosity.


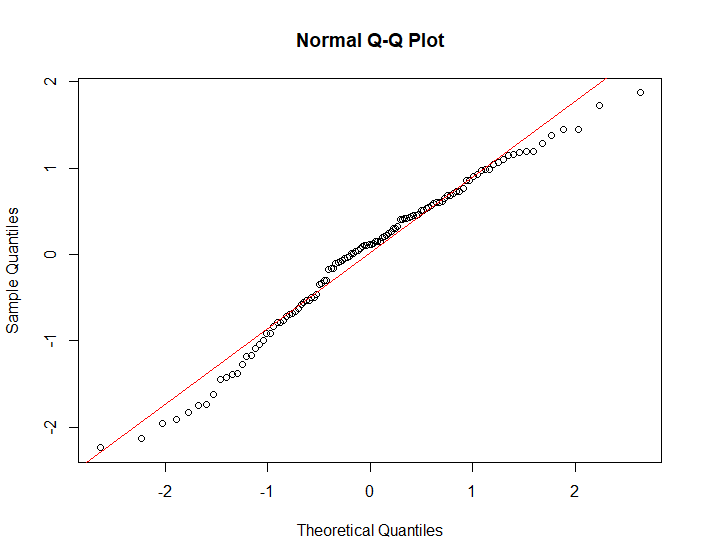


Figure S6: Residuals of best genetic models for GINs plotted against fitted values for model sets (i) sMLH + DRB1 heterozygosity status (A), (ii) sMLH + DRB1*0114 alleles (B) and (iii) sMLH + DRB1genotypes (C).


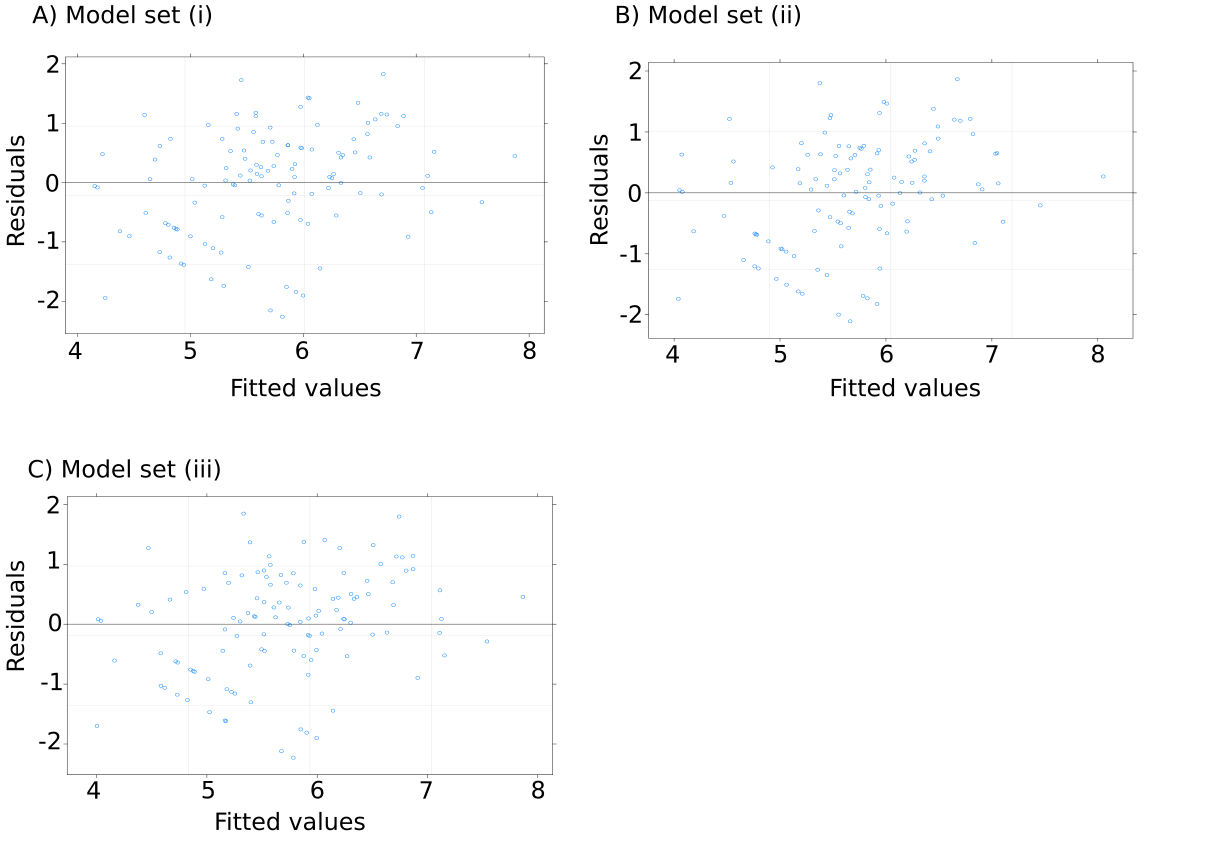

Supplement: Supplementary file 2 — Additional file 2. Normality tests of non-genetic and genetic models. [file 12898_2019_228_MOESM2_ESM.docx]
