# Supplementary material for: Both candidate gene and neutral genetic diversity correlate with parasite resistance in female Mediterranean mouflon
Source: BMC Ecol. 2019 Mar 5;19:12. doi: 10.1186/s12898-019-0228-x (PMC6402107; doi:10.1186/s12898-019-0228-x)
Supplement: Supplementary file 3 — Additional file 3. Variance Inflation Factors values for genetic models and tests of the presence of sMLH quadratic effects on FOC and FEC. [file 12898_2019_228_MOESM3_ESM.docx]

***Both candidate gene and neutral genetic diversity correlate with parasite resistance in female Mediterranean mouflon***

Elodie Portanier^1, 2, 3^, Mathieu Garel^2^, Sébastien Devillard^1^, Daniel Maillard^2^, Jocelyn Poissant^4^, Maxime Galan^5^, Slimania Benabed^3^, Marie-Thérèse Poirel^3^, Jeanne Duhayer^2^, Christian Itty^2^ and Gilles Bourgoin^1, 3^

*^1^Univ Lyon, Université Claude Bernard Lyon 1, CNRS, Laboratoire de Biométrie et Biologie Évolutive, F-69100, Villeurbanne, France.*

^2^*Office National de la Chasse et de la Faune Sauvage, Unité Ongulés Sauvages, 5 allée de Bethléem, Z.I. Mayencin F-38610, Gières, France.*

^3^*Université de Lyon, VetAgro Sup, Campus Vétérinaire de Lyon, 1 Avenue Bourgelat, BP 83 F-69280, Marcy l’Etoile, France.*

*^4^Department of Ecosystem and Public Health, University of Calgary, Calgary, Canada.*

*^5^CBGP, INRA, CIRAD, IRD, Montpellier SupAgro, Université de Montpellier, F-34980, Montferrier sur Lez, France.*

**Correspondence:** Elodie Portanier, Université Claude Bernard Lyon 1, CNRS, Laboratoire de Biométrie et Biologie Évolutive, 69100, Villeurbanne, France, Fax: +33 4 72 43 13 88, E-mail: elodie.portanier@gmail.com

Additional file 3

Table S4: Variables used in genetic mixed-effects linear models and relative Variance Inflation Factors values (VIF) for both FEC and FOC in the three model sets: (i) sMLH + DRB1 heterozygosity status (H_DRB1), (ii) sMLH + presence of specific DRB1 alleles (R1, R2 and R3, for *0324, *07012 * and *0114, respectively) and (iii) sMLH + DRB1genotypes (G_DRB1). SMI stands for the body condition, sMLH is the multilocus heterozygosity, age is the age class of individuals, Julian day is the Julian day of sampling and time lapse represent the time elapsed between sampling and coproscopic analyses. Non-genetic terms were retained in the first step of the inferential approach (see main text). All models included the individual identity and the year of sampling as random effects.

| **FOC** | | | | | | | |  | | |  | | | |  | | |  | | | | |  | | | | |  | | | | |  | | | | |  | | |
| --- | --- | --- | --- | --- | --- | --- | --- | --- | --- | --- | --- | --- | --- | --- | --- | --- | --- | --- | --- | --- | --- | --- | --- | --- | --- | --- | --- | --- | --- | --- | --- | --- | --- | --- | --- | --- | --- | --- | --- | --- |
| *model set (i)* | |  | | | | | |  | | |  | | | |  | | |  | | | | |  | | | | |  | | | | |  | | | | |  | | |
| SMI  1.02 | Age-class 2 | | | | Age-class 3 | | | | | | | Time lapse | | | | | Julian day | | | | | H_DRB1 | | | | | sMLH | | | | sMLH² | | | | |  |  | |  |  |
|  | 1.06 | | | | 1.09 | | | | | | | 1.45 | | | | | 1.43 | | | | | 1.05 | | | | | 1.02 | | | | 1.04 | | | | |  |  | |  |  |
| *model set (ii)* | |  | | | | | |  | | |  | | | |  | | |  | | | | |  | | | | |  | | | | |  | | | | |  | | |
| SMI  1.05 | Age-class 2 | | | | | Age-class 3 | | | | | | | Time lapse | | | | Julian day | | | R1 | | | | R2 | | | R3 | | sMLH | | | | | sMLH² | | | | | |  |
|  | 1.06 | | | | | 1.12 | | | | | | | 1.47 | | | | 1.45 | | | 1.49 | | | | 1.64 | | | 1.27 | | 1.05 | | | | | 1.13 | | | | | |  |
| *model set (iii)* | |  | | | | | |  | | |  | | | |  | | |  | | | | |  | | | | |  | | | | |  | | | | |  | | |
| SMI  1.06 | Age-class 2 | Age-class 3 | | | | | Time lapse | | | Julian day | | | | G_DRB1 B | | | G_DRB1 C | | G_DRB1 D | | | | | | G_DRB1 E | | | | | sMLH | | | | | sMLH² | | | | | |
|  | 1.07 | 1.12 | | | | | 1.48 | | | 1.46 | | | | 1.35 | | | 1.25 | | 1.25 | | | | | | 1.17 | | | | | 1.05 | | | | | 1.22 | | | | | |
| **FEC** | |  | | | | | |  | | |  | | | |  | | |  | | | | |  | | | | |  | | | | |  | | | | |  | | |
| *model set (i)* | |  | | | | | |  | | |  | | | |  | | |  | | | | |  | | | | |  | | | | |  | | | | |  | | |
| SMI  1.01 | H_DRB1 | | sMLH | | | | | | sMLH² | | | | |  |  | | |  | | | | |  | | | | |  | | | | |  | | | | |  | | |
|  | 1.02 | | 1.00 | | | | | | 1.01 | | | | |  |  | | |  | | | | |  | | | | |  | | | | |  | | | | |  | | |
| *model set (ii)* | |  | | | | | |  | | |  | | | |  | | |  | | | | |  | | | | |  | | | | |  | | | | |  | | |
| SMI | R1 | R2 | | | | | | R3 | | | sMLH | | | | sMLH² | | |  | | | | |  | | | | |  | | | | |  | | | | |  | | |
| 1.02 | 1.47 | 1.52 | | | | | | 1.25 | | | 1.03 | | | | 1.08 | | |  | | | | |  | | | | |  | | | | |  | | | | |  | | |
| *model set (iii)* | |  | | | | | |  | | |  | | | |  | | |  | | | | |  | | | | |  | | | | |  | | | | |  | | |
| SMI  1.04 | G_DRB1 B | | | G_DRB1 C | | | | | | | G_DRB1 D | | | | | G_DRB1 E | | | | | sMLH | | | | | sMLH² | | | | |  |  |  | | | | |  | | |
|  | 1.29 | | | 1.25 | | | | | | | 1.23 | | | | | 1.11 | | | | | 1.03 | | | | | 1.18 | | | | |  |  |  | | | | |  | | |

Table S5: Model selection of mixed-effects models based on corrected Akaike’s Information Criterion (AICc) for testing the effects of quadratic terms of sMLH on parasite resistance as measured by FOC and FEC in the three model sets: (i) sMLH + DRB1 heterozygosity status (H_DRB1), (ii) sMLH + presence of specific DRB1 alleles (R1, R2 and R3, for *0324, *07012 * and *0114, respectively) and (iii) sMLH + DRB1genotypes (G_DRB1). *NG* stands for the non-genetic variables retained from the first step of the modeling approach (see main text and Additional file 1). *NG* included body condition, Julian day of sampling, age class of individuals and the time elapsed between sampling and coproscopic analyses for FOC. For FEC, NG only included body condition. All models included the individual identity and the year of sampling as random effects.

| Response variable | Component of the model | AICc |
| --- | --- | --- |
| FOC | *NG* + H_DRB1 + sMLH | 383.26 |
|  | *NG* + H_DRB1 + sMLH+ sMLH² | 382.01 |
|  | *NG* + R1 + R2 + R3 + sMLH | 386.38 |
|  | *NG* + R1 + R2 + R3 + sMLH+ sMLH² | 386.09 |
|  | *NG* + G_DRB1 + sMLH | 388.65 |
|  | *NG* + G_DRB1 + sMLH+ sMLH² | 388.71 |
| FEC | *NG* + H_DRB1 + sMLH | 384.03 |
|  | *NG* + H_DRB1 + sMLH+ sMLH² | 378.34 |
|  | *NG* + R1 + R2 + R3 + sMLH | 384.52 |
|  | *NG* + R1 + R2 + R3 + sMLH+ sMLH² | 381.07 |
|  | *NG* + G_DRB1 + sMLH | 386.86 |
|  | *NG* + G_DRB1 + sMLH+ sMLH² | 383.27 |
