# Supplementary material for: Both candidate gene and neutral genetic diversity correlate with parasite resistance in female Mediterranean mouflon
Source: BMC Ecol. 2019 Mar 5;19:12. doi: 10.1186/s12898-019-0228-x (PMC6402107; doi:10.1186/s12898-019-0228-x)
Supplement: Supplementary file 4 — Additional file 4. R code used for statistical analyses. [file 12898_2019_228_MOESM4_ESM.docx]

***Both candidate gene and neutral genetic diversity correlate with parasite resistance in female Mediterranean mouflon***

Elodie Portanier^1, 2, 3^, Mathieu Garel^2^, Sébastien Devillard^1^, Daniel Maillard^2^, Jocelyn Poissant^4^, Maxime Galan^5^, Slimania Benabed^3^, Marie-Thérèse Poirel^3^, Jeanne Duhayer^2^, Christian Itty^2^ and Gilles Bourgoin^1, 3^

*^1^Univ Lyon, Université Claude Bernard Lyon 1, CNRS, Laboratoire de Biométrie et Biologie Évolutive, F-69100, Villeurbanne, France.*

^2^*Office National de la Chasse et de la Faune Sauvage, Unité Ongulés Sauvages, 5 allée de Bethléem, Z.I. Mayencin F-38610, Gières, France.*

^3^*Université de Lyon, VetAgro Sup, Campus Vétérinaire de Lyon, 1 Avenue Bourgelat, BP 83 F-69280, Marcy l’Etoile, France.*

*^4^Department of Ecosystem and Public Health, University of Calgary, Calgary, Canada.*

*^5^CBGP, INRA, CIRAD, IRD, Montpellier SupAgro, Université de Montpellier, F-34980, Montferrier sur Lez, France.*

**Correspondence:** Elodie Portanier, Université Claude Bernard Lyon 1, CNRS, Laboratoire de Biométrie et Biologie Évolutive, 69100, Villeurbanne, France, Fax: +33 4 72 43 13 88, E-mail: elodie.portanier@gmail.com

Additional file 4

###########################

#### Libraries and functions ####

###########################

library(inbreedR)

library(ade4)

library(mgcv)

library(MuMIn)

library(lme4)

library(lmer data)

library(gridExtra)

library(ggplot2)

library(plyr)

library(effects)

library(plotrix)

library(visreg)

## VIF calculation for lmer models (Frank 2011)

## https://github.com/aufrank/R-hacks/blob/master/mer-utils.R

vif.mer <- function (fit) {

## adapted from rms::vif

v <- vcov(fit)

nam <- names(fixef(fit))

## exclude intercepts

ns <- sum(1 * (nam == "Intercept" | nam == "(Intercept)"))

if (ns > 0) {

v <- v[-(1:ns), -(1:ns), drop = FALSE]

nam <- nam[-(1:ns)] }

d <- diag(v)^0.5

v <- diag(solve(v/(d %o% d)))

names(v) <- nam

v }

## delta AIC calculation

deltaAIC <- function(x)

{

if (!(any(rank(x)==1:length(x))))

stop("x should be sorted")

deltaAIC <- x-x[1]

return(deltaAIC)

}

## SMI calculation (see Peig & Green 2009, 2010)

## M = body mass

## L = body length

## L0 = arithmetic mean

ICC <- function(M,L,L0){ #,FAT

## SMI (Scaled Mass Index)

bsma <- coef(lm(log(M)~log(L)))[[2]]/cor(log(M),log(L))

Mi <- M*(L0/L)^bsma

## Residual index

#Ri <- residuals(lm(log(M)~log(L)))

return(Mi)

# cbind.data.frame(Mi,Ri)

}

#############

#### Data ####

#############

## log of Eimeria spp. counts

data$eimeriatr=log(data$eimeria)

## log of GINs counts

data$stronglestr=log(data$strongles+10)

##############################

####### Non-genetic models #######

##############################

## iccsc = scaled SMI

## Sec = socio-spatial unit of females

## agecl = age class

## intersc = scaled timelaps between sample and coproscopy

## daysc = scaled day of sampling (julian date)

## id = individual identity

## yr = year of sampling

## code is here provided for GINs but the same procedure was applied for Eimeria spp.

## searching for quadratic effects

# Complete non genetic model without quadratic term

AICc(lmer(data = data , stronglestr ~ iccsc + Sec + agecl+ intersc + daysc + (1|id)+ (1|yr),na.action = "na.fail",REML=F))

## Quadratic SMI

AICc(lmer(data = data , stronglestr ~ Sec + agecl+ intersc + daysc + poly(iccsc,2) +(1|id)+ (1|yr),na.action = "na.fail",REML=F))

## Quadratic day of sampling

AICc(lmer(data = data , stronglestr ~ iccsc + Sec + agecl + intersc + poly(daysc,2) +(1|id)+ (1|yr),na.action = "na.fail",REML=F))

## Quadratic timelaps between sample and coproscopy

AICc(lmer(data = data , stronglestr ~ iccsc + Sec + agecl + daysc + poly(intersc,2) +(1|id)+ (1|yr),na.action = "na.fail",REML=F))

## VIF calculation

round(vif.mer(lmer(data = data , stronglestr ~ iccsc + Sec + agecl+ intersc + daysc + (1|id)+ (1|yr),na.action = "na.fail",REML=F)),2)

## identifying the best non genetic model for Eimeria spp. (no quadratic terms for non genetic variables)

dredge(lmer(data = data , stronglestr ~ Sec + iccsc + intersc + agecl + daysc + (1|id)+ (1|yr),na.action = "na.fail",REML=F))

## reduced best model

mod1 <- lmer(data = data, stronglestr ~ iccsc + (1|id)+ (1|yr) ,na.action = "na.fail",REML=T)

summary(mod1)

## residuals normality assessment

qqnorm(residuals(mod1))

qqline(residuals(mod1), col = 2)

shapiro.test(residuals(mod1))

## goodness of fit

r.squaredGLMM(mod1)

## figures using visreg

visreg(mod1,"iccsc", type="conditional", trans=exp,line=list(col="black"))

##########################

####### Genetic models #######

##########################

## R1, R2 and R3 = the 3 alleles of DRB1 gene identified (see main article)

## HDRB = Heterozygosity at DRB1 locus (1 = heterozygous, 0 = homozygous)

## G_DRB1 = DRB1 gentoype of individuals

## searching for quadratic effects

# Complete genetic model without quadratic term

AICc(lmer(data = data , stronglestr ~ iccsc + R1 + R2 + R3 + MLHsc + (1|id) + (1|yr),na.action = "na.fail",REML=F))

AICc(lmer(data = data , stronglestr ~ iccsc + HDRB + MLHsc + (1|id) + (1|yr),na.action = "na.fail",REML=F))

AICc(lmer(data = data , stronglestr ~ iccsc + G_DRB1 + MLHsc + (1|id) + (1|yr),na.action = "na.fail",REML=F))

## Quadratic sMLH

AICc(lmer(data = data , stronglestr ~iccsc + intersc + daysc +R1 + R2 + R3 + poly(MLHsc,2)+ (1|id) + (1|yr),na.action = "na.fail",REML=F))

AICc(lmer(data = data , stronglestr ~iccsc + HDRB + poly(MLHsc,2)+ (1|id) + (1|yr),na.action = "na.fail",REML=F))

AICc(lmer(data = data , stronglestr ~iccsc + G_DRB1 + poly(MLHsc,2)+ (1|id) + (1|yr),na.action = "na.fail",REML=F))

## VIF calculation

round(vif.mer(lmer(data = data , stronglestr ~ iccsc + R1 + R2 + R3 + poly(MLHsc,2) + (1|id) + (1|yr))),2)

round(vif.mer(lmer(data = data , stronglestr ~ iccsc + HDRB + poly(MLHsc,2) + (1|id) + (1|yr))) ,2)

round(vif.mer(lmer(data = data , stronglestr ~ iccsc + G_DRB1 + poly(MLHsc,2) + (1|id) + (1|yr))),2)

## Here, since we have to keep all the non genetic variable in the models, we don't use the dredge function

## Selection of the best genetic model in each model set (i, ii, iii, see main article)

M <- list()

M[[1]]=lmer(data = data , stronglestr ~ iccsc + (1|id) + (1|yr),na.action = "na.fail",REML=F)

M[[2]]=lmer(data = data , stronglestr ~ iccsc + HDRB + (1|id) + (1|yr),na.action = "na.fail",REML=F)

M[[3]]=lmer(data = data , stronglestr ~ iccsc + HDRB + poly(MLHsc,2) + (1|id) + (1|yr),na.action = "na.fail",REML=F)

M[[4]]=lmer(data = data , stronglestr ~ iccsc + G_DRB1 + (1|id) + (1|yr),na.action = "na.fail",REML=F)

M[[5]]=lmer(data = data , stronglestr ~ iccsc + G_DRB1 + poly(MLHsc,2) + (1|id) + (1|yr),na.action = "na.fail",REML=F)

M[[6]]=lmer(data = data , stronglestr ~ iccsc + R1 + (1|id) + (1|yr),na.action = "na.fail",REML=F)

M[[7]]=lmer(data = data , stronglestr ~ iccsc + R2 + (1|id) + (1|yr),na.action = "na.fail",REML=F)

M[[8]]=lmer(data = data , stronglestr ~ iccsc + R3 + (1|id) + (1|yr),na.action = "na.fail",REML=F)

M[[9]]=lmer(data = data , stronglestr ~ iccsc + R1 + poly(MLHsc,2) + (1|id) + (1|yr),na.action = "na.fail",REML=F)

M[[10]]=lmer(data = data , stronglestr ~ iccsc + R2 + poly(MLHsc,2) + (1|id) + (1|yr),na.action = "na.fail",REML=F)

M[[11]]=lmer(data = data , stronglestr ~ iccsc + R3 + poly(MLHsc,2) + (1|id) + (1|yr),na.action = "na.fail",REML=F)

M[[12]]=lmer(data = data , stronglestr ~ iccsc + R1 + R2 + (1|id) + (1|yr),na.action = "na.fail",REML=F)

M[[13]]=lmer(data = data , stronglestr ~ iccsc + R1 + R3 + (1|id) + (1|yr),na.action = "na.fail",REML=F)

M[[14]]=lmer(data = data , stronglestr ~ iccsc + R3 + R2 + (1|id) + (1|yr),na.action = "na.fail",REML=F)

M[[15]]=lmer(data = data , stronglestr ~ iccsc + R1 + R2 + poly(MLHsc,2) + (1|id) + (1|yr),na.action = "na.fail",REML=F)

M[[16]]=lmer(data = data , stronglestr ~ iccsc + R1 + R3 + poly(MLHsc,2) + (1|id) + (1|yr),na.action = "na.fail",REML=F)

M[[17]]=lmer(data = data , stronglestr ~ iccsc + R3 + R2 + poly(MLHsc,2) + (1|id) + (1|yr),na.action = "na.fail",REML=F)

M[[18]]=lmer(data = data , stronglestr ~ iccsc + R3 + R2 + R1 + (1|id) + (1|yr),na.action = "na.fail",REML=F)

M[[19]]=lmer(data = data , stronglestr ~ iccsc + R3 + R2 + R1 + poly(MLHsc,2) + (1|id) + (1|yr),na.action = "na.fail",REML=F)

M[[20]]=lmer(data = data , stronglestr ~ iccsc + poly(MLHsc,2) + (1|id) + (1|yr),na.action = "na.fail",REML=F)

modnames=c("non_genet", "HDRB","MLH²_HDRB","G_DRB1","MLH²_G_DRB1","R1","R2","R3","MLH²_R1","MLH²_R2","MLH²_R3","R1_R2","R1_R3","R2_R3",

"MLH²_R1_R2","MLH²_R1_R3","MLH²_R2_R3","R1_R2_R3","MLH²_R1_R2_R3","MLH²")

res.selEF<-cbind(modnames, rbind.fill(lapply(M,function(x){as.data.frame(t(c(npar = attr(logLik(x), "df"), AICc = AICc(x), fixef(x))),

stringsAsFactors=FALSE)})))

res.selEF<-res.selEF[order(res.selEF$AICc),]

res.selEF$Delta.AICc <- deltaAIC(res.selEF$AICc)

res.selEF$weight <- Weights(res.selEF$AICc)

res.selEF[,3:ncol(res.selEF)] <- round(res.selEF[,3:ncol(res.selEF)], 3)

res.selEF

## reduced best models

mod1 <- lmer(data = data , stronglestr ~ iccsc + G_DRB1 + poly(MLHsc,2) + (1|id) + (1|yr),na.action = "na.fail",REML=T)

summary(mod1)

mod2 <-lmer(data = data , stronglestr ~ iccsc + HDRB + poly(MLHsc,2) + (1|id) + (1|yr),na.action = "na.fail",REML=T)

summary(mod2)

mod3 <- lmer(data = data , stronglestr ~ iccsc + R3 + poly(MLHsc,2) + (1|id) + (1|yr),na.action = "na.fail",REML=T)

summary(mod3)

## residuals normality assessment

qqnorm(residuals(mod1))

qqline(residuals(mod1), col = 2)

shapiro.test(residuals(mod1))

qqnorm(residuals(mod2))

qqline(residuals(mod2), col = 2)

shapiro.test(residuals(mod2))

qqnorm(residuals(mod3))

qqline(residuals(mod3), col = 2)

shapiro.test(residuals(mod3))

## goodness of fit

r.squaredGLMM(mod1)

r.squaredGLMM(mod2)

r.squaredGLMM(mod3)

## figures using visreg

par(mfrow=c(1,3))

mod=lmer(data = data, stronglestr ~ iccsc + HDRB + MLHsc + MLHsc^2+ (1|id) + (1|yr) ,na.action = "na.fail",REML=T)

visreg(mod,"HDRB", type="conditional", trans=exp,partial=F,line=list(col="black"))

# visreg(mod,"iccsc", by="HDRB", overlay=TRUE)

mod=lmer(data = data, stronglestr ~ iccsc + R3 +MLHsc + MLHsc^2+ (1|id) + (1|yr) ,na.action = "na.fail",REML=T)

visreg(mod,"R3", type="conditional", trans=exp,line=list(col="black"))

mod=lmer(data = data, stronglestr ~ iccsc + G_DRB1 +MLHsc + MLHsc^2+ (1|id) + (1|yr) ,na.action = "na.fail",REML=T)

visreg(mod,"G_DRB1", type="conditional", trans=exp,line=list(col="black"))

## plot of the quadratic relationship

par(mfrow=c(1,3))

mod=lmer(data = data, stronglestr ~ iccsc + HDRB + poly(MLHsc,2) +(1|id) + (1|yr) ,na.action = "na.fail",REML=T)

visreg(mod,"MLHsc",trans=exp,line=list(col="black"),ylim = c(0, 2200),rug=2)

mod=lmer(data = data, stronglestr ~ iccsc + R3 + poly(MLHsc,2) +(1|id) + (1|yr) ,na.action = "na.fail",REML=T)

visreg(mod,"MLHsc",trans=exp,line=list(col="black"),ylim = c(0, 2200),rug=T)

mod=lmer(data = data, stronglestr ~ iccsc + G_DRB1 + poly(MLHsc,2) +(1|id) + (1|yr) ,na.action = "na.fail",REML=T)

visreg(mod,"MLHsc",trans=exp,line=list(col="black"),ylim = c(0, 2200),rug=T)

###########################################################

########### Discriminating between local and global effects ###########

###########################################################

## Following the procedure of Szulkin et al 2010

## effet_loc = table of genotypes by loci, coded as 1 if the individual is heterozygous and 0 if homozygous

## l1 - l16 = loci

## when missing data are present, we replaced NA by the mean value of heterozygotie at the locus considered

effet_loc[is.na(effet_loc$l1)%in%c("TRUE"),]$l1=mean(effet_loc[!is.na(effet_loc$l1)%in%c("TRUE"),]$l1)

effet_loc[is.na(effet_loc$l2)%in%c("TRUE"),]$l2=mean(effet_loc[!is.na(effet_loc$l2)%in%c("TRUE"),]$l2)

effet_loc[is.na(effet_loc$l3)%in%c("TRUE"),]$l3=mean(effet_loc[!is.na(effet_loc$l3)%in%c("TRUE"),]$l3)

effet_loc[is.na(effet_loc$l4)%in%c("TRUE"),]$l4=mean(effet_loc[!is.na(effet_loc$l4)%in%c("TRUE"),]$l4)

effet_loc[is.na(effet_loc$l5)%in%c("TRUE"),]$l5=mean(effet_loc[!is.na(effet_loc$l5)%in%c("TRUE"),]$l5)

effet_loc[is.na(effet_loc$l6)%in%c("TRUE"),]$l6=mean(effet_loc[!is.na(effet_loc$l6)%in%c("TRUE"),]$l6)

effet_loc[is.na(effet_loc$l7)%in%c("TRUE"),]$l7=mean(effet_loc[!is.na(effet_loc$l7)%in%c("TRUE"),]$l7)

effet_loc[is.na(effet_loc$l8)%in%c("TRUE"),]$l8=mean(effet_loc[!is.na(effet_loc$l8)%in%c("TRUE"),]$l8)

effet_loc[is.na(effet_loc$l9)%in%c("TRUE"),]$l9=mean(effet_loc[!is.na(effet_loc$l9)%in%c("TRUE"),]$l9)

effet_loc[is.na(effet_loc$l10)%in%c("TRUE"),]$l10=mean(effet_loc[!is.na(effet_loc$l10)%in%c("TRUE"),]$l10)

effet_loc[is.na(effet_loc$l11)%in%c("TRUE"),]$l11=mean(effet_loc[!is.na(effet_loc$l11)%in%c("TRUE"),]$l11)

effet_loc[is.na(effet_loc$l12)%in%c("TRUE"),]$l12=mean(effet_loc[!is.na(effet_loc$l12)%in%c("TRUE"),]$l12)

effet_loc[is.na(effet_loc$l13)%in%c("TRUE"),]$l13=mean(effet_loc[!is.na(effet_loc$l13)%in%c("TRUE"),]$l13)

effet_loc[is.na(effet_loc$l14)%in%c("TRUE"),]$l14=mean(effet_loc[!is.na(effet_loc$l14)%in%c("TRUE"),]$l14)

effet_loc[is.na(effet_loc$l15)%in%c("TRUE"),]$l15=mean(effet_loc[!is.na(effet_loc$l15)%in%c("TRUE"),]$l15)

effet_loc[is.na(effet_loc$l16)%in%c("TRUE"),]$l16=mean(effet_loc[!is.na(effet_loc$l16)%in%c("TRUE"),]$l16)

effet_loc$l1=as.factor(effet_loc$l1)

effet_loc$l2=as.factor(effet_loc$l2)

effet_loc$l3=as.factor(effet_loc$l3)

effet_loc$l4=as.factor(effet_loc$l4)

effet_loc$l5=as.factor(effet_loc$l5)

effet_loc$l6=as.factor(effet_loc$l6)

effet_loc$l7=as.factor(effet_loc$l7)

effet_loc$l8=as.factor(effet_loc$l8)

effet_loc$l9=as.factor(effet_loc$l9)

effet_loc$l10=as.factor(effet_loc$l10)

effet_loc$l11=as.factor(effet_loc$l11)

effet_loc$l12=as.factor(effet_loc$l12)

effet_loc$l13=as.factor(effet_loc$l13)

effet_loc$l14=as.factor(effet_loc$l14)

effet_loc$l15=as.factor(effet_loc$l15)

effet_loc$l16=as.factor(effet_loc$l16)

## "global" model

## loc 1 = table merging effet_loc and data

modGs=lm(data = loc1 ,stronglestr~ iccsc + poly(MLHsc,2) , na.action = "na.fail")

## "local" model

modLs=lm(data = loc1 , stronglestr~ iccsc + l1 + l2 + l3 + l4+ l5 + l6 + l7 + l8 + l9 + l10 + l11 + l12 + l13 + l14 + l15 + l16, na.action = "na.fail")

## F-ratio test to determine if the “local” model explains more variance than the “global” model

anova(modGs,modLs)

## Following the standardized procedure of Szulkin et al 2010

effet_loc[is.na(effet_loc$l1)%in%c("TRUE"),]$l1=mean(effet_loc[!is.na(effet_loc$l1)%in%c("TRUE"),]$l1)

effet_loc[is.na(effet_loc$l2)%in%c("TRUE"),]$l2=mean(effet_loc[!is.na(effet_loc$l2)%in%c("TRUE"),]$l2)

effet_loc[is.na(effet_loc$l3)%in%c("TRUE"),]$l3=mean(effet_loc[!is.na(effet_loc$l3)%in%c("TRUE"),]$l3)

effet_loc[is.na(effet_loc$l4)%in%c("TRUE"),]$l4=mean(effet_loc[!is.na(effet_loc$l4)%in%c("TRUE"),]$l4)

effet_loc[is.na(effet_loc$l5)%in%c("TRUE"),]$l5=mean(effet_loc[!is.na(effet_loc$l5)%in%c("TRUE"),]$l5)

effet_loc[is.na(effet_loc$l6)%in%c("TRUE"),]$l6=mean(effet_loc[!is.na(effet_loc$l6)%in%c("TRUE"),]$l6)

effet_loc[is.na(effet_loc$l7)%in%c("TRUE"),]$l7=mean(effet_loc[!is.na(effet_loc$l7)%in%c("TRUE"),]$l7)

effet_loc[is.na(effet_loc$l8)%in%c("TRUE"),]$l8=mean(effet_loc[!is.na(effet_loc$l8)%in%c("TRUE"),]$l8)

effet_loc[is.na(effet_loc$l9)%in%c("TRUE"),]$l9=mean(effet_loc[!is.na(effet_loc$l9)%in%c("TRUE"),]$l9)

effet_loc[is.na(effet_loc$l10)%in%c("TRUE"),]$l10=mean(effet_loc[!is.na(effet_loc$l10)%in%c("TRUE"),]$l10)

effet_loc[is.na(effet_loc$l11)%in%c("TRUE"),]$l11=mean(effet_loc[!is.na(effet_loc$l11)%in%c("TRUE"),]$l11)

effet_loc[is.na(effet_loc$l12)%in%c("TRUE"),]$l12=mean(effet_loc[!is.na(effet_loc$l12)%in%c("TRUE"),]$l12)

effet_loc[is.na(effet_loc$l13)%in%c("TRUE"),]$l13=mean(effet_loc[!is.na(effet_loc$l13)%in%c("TRUE"),]$l13)

effet_loc[is.na(effet_loc$l14)%in%c("TRUE"),]$l14=mean(effet_loc[!is.na(effet_loc$l14)%in%c("TRUE"),]$l14)

effet_loc[is.na(effet_loc$l15)%in%c("TRUE"),]$l15=mean(effet_loc[!is.na(effet_loc$l15)%in%c("TRUE"),]$l15)

effet_loc[is.na(effet_loc$l16)%in%c("TRUE"),]$l16=mean(effet_loc[!is.na(effet_loc$l16)%in%c("TRUE"),]$l16)

# hist=(hi)/(1-himoy*(1+g2))

# hi =1 for an heterozygote,0 for an homozygote),

# the sample average= hbarre

# hbarre=sum(loc2$l1)/length(loc2$l1)

h1=c()

g2=0.008 ## calculated using RMES (David et al. 2007)

## loc2 = merge(effet_loc,data)

## fichier = individual identity

for (i in loc2$fichier)

{

h1=as.data.frame(rbind(h1, c(i,cbind((loc2[loc2$fichier==i,]$l1)/(1-((sum(loc2$l1)/length(loc2$l1))*(1+g2))),

(loc2[loc2$fichier==i,]$l2)/(1-((sum(loc2$l2)/length(loc2$l2))*(1+g2))),

(loc2[loc2$fichier==i,]$l3)/(1-((sum(loc2$l3)/length(loc2$l3))*(1+g2))),

(loc2[loc2$fichier==i,]$l4)/(1-((sum(loc2$l4)/length(loc2$l4))*(1+g2))),

(loc2[loc2$fichier==i,]$l5)/(1-((sum(loc2$l5)/length(loc2$l5))*(1+g2))),

(loc2[loc2$fichier==i,]$l6)/(1-((sum(loc2$l6)/length(loc2$l6))*(1+g2))),

(loc2[loc2$fichier==i,]$l7)/(1-((sum(loc2$l7)/length(loc2$l7))*(1+g2))),

(loc2[loc2$fichier==i,]$l8)/(1-((sum(loc2$l8)/length(loc2$l8))*(1+g2))),

(loc2[loc2$fichier==i,]$l9)/(1-((sum(loc2$l9)/length(loc2$l9))*(1+g2))),

(loc2[loc2$fichier==i,]$l10)/(1-((sum(loc2$l10)/length(loc2$l10))*(1+g2))),

(loc2[loc2$fichier==i,]$l11)/(1-((sum(loc2$l11)/length(loc2$l11))*(1+g2))),

(loc2[loc2$fichier==i,]$l12)/(1-((sum(loc2$l12)/length(loc2$l12))*(1+g2))),

(loc2[loc2$fichier==i,]$l13)/(1-((sum(loc2$l13)/length(loc2$l13))*(1+g2))),

(loc2[loc2$fichier==i,]$l14)/(1-((sum(loc2$l14)/length(loc2$l14))*(1+g2))),

(loc2[loc2$fichier==i,]$l15)/(1-((sum(loc2$l15)/length(loc2$l15))*(1+g2))),

(loc2[loc2$fichier==i,]$l16)/(1-((sum(loc2$l16)/length(loc2$l16))*(1+g2)))))))

}

colnames(h1)=c("fichier","l1","l2","l3","l4","l5","l6","l7","l8","l9","l10","l11","l12","l13","l14","l15","l16")

## loc3 = cbind(loc2,h1)

loc3$l1=as.factor(loc3$l1)

loc3$l2=as.factor(loc3$l2)

loc3$l3=as.factor(loc3$l3)

loc3$l4=as.factor(loc3$l4)

loc3$l5=as.factor(loc3$l5)

loc3$l6=as.factor(loc3$l6)

loc3$l7=as.factor(loc3$l7)

loc3$l8=as.factor(loc3$l8)

loc3$l9=as.factor(loc3$l9)

loc3$l10=as.factor(loc3$l10)

loc3$l11=as.factor(loc3$l11)

loc3$l12=as.factor(loc3$l12)

loc3$l13=as.factor(loc3$l13)

loc3$l14=as.factor(loc3$l14)

loc3$l15=as.factor(loc3$l15)

loc3$l16=as.factor(loc3$l16)

## "global" model

modGs=lm(data = loc3, stronglestr ~ iccsc + poly(MLHsc,2),na.action = "na.fail")

## "local" model

modLs=lm(data = loc3, stronglestr ~ iccsc + poly(MLHsc,2) + l1 + l2 + l3 + l4 + l5 + l6 + l7 + l8 + l9 + l10 + l11 + l12 + l13 + l14 + l15 + l16, na.action = "na.fail")

## F-ratio test to determine if the “local” model explains more variance than the “global” model

anova(modGs,modLs)
